# Impact of COVID-19 on testicular function: a systematic review and meta-analysis

**DOI:** 10.1007/s12020-024-03705-7

**Published:** 2024-02-12

**Authors:** Rossella Cannarella, Marta Marino, Andrea Crafa, Vincenzo Bagnara, Sandro La Vignera, Rosita A. Condorelli, Aldo E. Calogero

**Affiliations:** 1 Department of Clinical and Experimental Medicine, University of Catania, Catania, Italy; 2 Glickman Urological & Kidney Institute, Cleveland Clinic Foundation, Cleveland, OH USA; 3 Pediatric Surgery Unit, Policlinic G.B. Morgagni, Catania, Italy

**Keywords:** SARS-CoV2, COVID-19, Testicular function, Infertility, Testosterone

## Abstract

**Introduction:**

Studies investigating the effects of SARS-CoV-2 on male reproductive function are few and heterogeneous, and results are often conflicting. This systematic review and meta-analysis was carried out on studies conducted in men with active or anamnestic SARS-CoV-2 infection to evaluate its consequences on the male sex hormone profile and semen parameters.

**Materials and method:**

This meta-analysis follows the Preferred Reporting Items for Systematic Review and Meta-Analysis (PRISMA) protocols. PubMed, Scopus, Cochrane, and Embase databases were searched to identify relevant studies. We originally selected 3553 articles. After the eligibility phase, 16 articles met our inclusion criteria encompassing 11 case-control studies and 5 cohort studies (2 prospective and 3 retrospective studies). We performed the quantitative analysis with Comprehensive Meta-Analysis Software. Cochran-*Q* and heterogeneity (*I*^2^) indexes were used to assess statistical heterogeneity. Sensitivity analysis and publication bias tests were also performed.

**Results:**

Overall, 1250 patients with active or recent (up to 80 days before) COVID-19 infection and 1232 matched healthy controls were included. Sperm concentration, total sperm count, and total motility were significantly lower in patients compared with controls. Patients also showed lower levels of total testosterone and follicle-stimulating hormone, and higher levels of luteinizing hormone, 17β-estradiol, and prolactin compared with healthy controls. None of the included studies found the presence of SARS-CoV-2 mRNA in the semen of infected patients.

**Conclusion:**

The present systematic review and meta-analysis suggests the presence of an association between SARS-CoV-2 infection and primary testicular damage manifested with a picture of altered steroidogenesis and worsening spermatogenesis. The absence of the virus in the seminal fluid indicates a low possibility of sexual transmission of the infection to partners and offspring. However, our findings mostly show short-term follow-up, while few studies have considered the long-term consequences of the viral infection, thus further studies are needed to evaluate the long-term consequences on male reproductive health.

## Introduction

The severe acute respiratory syndrome Coronavirus 2 (SARS-CoV-2) epidemic broke out in China, in the city of Wuhan, in December 2019 [1]. The virus belongs to the *Coronaviridae* family and probably originated by spill-over from animal species. However, it shares many sequences with SARS-CoV1, a virus of the same family that gave rise to the Middle-East Respiratory Syndrome epidemic, which spread out from Saudi Arabia to the Middle East in 2012. SARS-CoV-2 was quickly shown to be extremely virulent and pathogenic, causing fatal interstitial pneumonia in a significant proportion of cases. On December 31st, 2019, WHO declared a pandemic status, as the virus had spread to every country of the world and every government moved to put in place collective safety and security regimes to halt the progression of the virus. To date, SARS-CoV-2 has generated 670 million cases and 6 million deaths worldwide (www.worldometers.info; https://www.worldometers.info/coronavirus/).

The interest of scientific research has shifted toward identifying the factors predisposing to infection and the severity of the disease. Current evidence has demonstrated that the mortality rate from COVID-19 depends on comorbidities, increasing progressively in patients with cardiovascular disease (CVD), diabetes mellitus, or hypertension [2]. Another conditioning factor is represented by age since only a small part of deaths is represented by the population under 50 years of age, while the greatest number of deaths is identified in the age groups over 80 years. These statistics appear to be present worldwide [3].

Epidemiological data of SARS-CoV-2 after the outbreak demonstrated a higher prevalence and severity in men, demonstrating a greater susceptibility to infection, a greater likelihood of a more aggressive course of the disease, and a greater prevalence of mortality [4, 5]. Despite a likely multi-factorial etiology, these earlier data may partially be explained by the expression of angiotensin-converting enzyme 2 (ACE2) and transmembrane serine protease 2 (TMPRSS2) in the male genital tract [6]. However, there is little evidence in vivo of orchitis linked to COVID-19. Additionally, few studies identified the SARS-CoV-2 in the semen and the possibility of sexual transmission is still a matter of concern [7]. This does not, however, rule out the possibility of unintended consequences on testicular function [8]. Though indirect damage brought on by inflammation, medications, and fever appears to be temporary, the virus does not appear to directly harm testicular function. However, fever has been shown to have negative effects on sperm quality [9].

To confirm these hypotheses, epidemiological data report that patients with hypogonadism seem to develop more complications [10]. Different suggestions have been proposed to explain these mechanisms. The first is based on the different hormonal milieu between the two sexes and implies a modulatory role of estrogens in the inflammatory state, which gives greater protection to women. However, testosterone also maintains an anti-inflammatory effect by reducing the cytokines most involved in systemic inflammation (IL-1, IL-6, TNFα). Indeed, several studies [11–16] have identified a worse prognosis in patients with known hypotestosteronemia, with a prolonged disease course, and longer stay in the Intensive Care Unit.

The virus is capable of infecting Leydig cells and it has been postulated that infected patients may develop hypogonadism [17]. Hypogonadism, and thus decreased total testosterone (TT) levels, can lead to destabilization of the immune response and endothelial dysfunction, resulting in impaired virus clearance and massive inflammatory response [18].

However, it is not yet known whether testosterone deficiency is a cause or a consequence of COVID-19 and whether it results from primary gonadal damage or hypothalamic-pituitary-testicular axis dysfunction [19]. It is readily apparent that COVID-19 can disrupt the hypothalamic-pituitary-gonadal (HPG) axis [20, 21]. The pathological state of steroidogenesis in the testis was reflected by variations in T levels, which were connected to the dysregulated levels of LH and FSH in COVID-19-affected individuals. In addition, the decreased T levels may result in altered spermatogenesis and erectile dysfunction, which support subfertility [22]. Higher levels of LH and FSH are indicative of adverse outcomes such as testicular injury [23]. In addition to hypothyroidism, dysregulation of the HPG axis has been linked to chronic renal illness, liver cirrhosis, and neurodegenerative senescence [24]. It must be said pharmacological interference cannot be excluded, since the mainstay of anti-inflammatory therapy is based on the administration of high doses of glucocorticoids which may affect the hypothalamus, resulting in suppression of pulsatile gonadotropin-releasing hormone (GnRH) secretion leading to central hypogonadism [25, 26].

Furthermore, the different hormonal milieu appears to play a role in the modulation of ACE2 expression [27]. ACE2 is part of the renin-angiotensin system; it is mainly located in the alveolar epithelium, although recent studies have identified a higher concentration in Leydig and Sertoli cells. SARS-CoV-2 has a high affinity for ACE2 which constitutes the entry gate of the virus into cells [6, 28, 29]. In addition, ACE2, as well as TMPRSS2, another protein implicated in the mechanism of virus entry into cells, has been found in spermatogonia and spermatids. Thus, a picture of viral testicular colonization is configured, probably capable of altering spermatogenesis [6, 30–36].

Also of particular concern is the viral localization in semen and the possible risk of its sexual transmission. Indeed, the virus has been isolated in the semen of infected patients. A review by Abdollahpour et al. confirmed sexual transmission of the virus in 16 out of 21 studies [37]. These implications acquire greater importance not only for the possible source of infection and extent of virus containment measures but also for the possible embryo and gestational damage that may result from spermatogonial infection. Indeed, the role of the virus in spermatogonia has yet to be investigated in scientific studies, as well as any consequences on conception and embryo health [38].

Numerous meta-analyses on the short-term impact of SARS-CoV-2 infection on testicular function have already been published. However, most studies only reported the effects on LH, FSH, and TT serum levels. Understanding the impact of the infection also on serum PRL, E_2_, and SHBG levels would be useful to have a more complete picture of the pathogenetic mechanisms through which the infection causes testicular dysfunction. Furthermore, not all studies included in previous meta-analyses consider positivity to SARS-CoV-2 on RT-PCR analysis of nasal or pharyngeal swabs and/or positive serology for IgA/IgA antibodies as the main criteria of COVID-19 diagnosis. Some studies, for example, only considered radiological images, making the diagnosis of SARS-CoV-2 infection uncertain.

With these premises, this systematic review and meta-analysis aims to comprehensively evaluate the effects of active or anamnestic SARS-CoV-2 infection on testicular function and, in particular, on the hypothalamic-pituitary-testicular axis and sperm parameters. Specifically, we wanted to analyze a wide range of hormones in patients in whom the diagnosis of COVID-19 was based on positivity to SARS-CoV-2 on RT-PCR analysis of nasal or pharyngeal swabs and/or positivity in serology for IgA/IgA antibodies.

## Material and methods

### Search strategy

This systematic review and meta-analysis was carried out according to the Preferred Reporting Items for Systematic Review and Meta-Analysis (PRISMA) Protocols. The literature search was performed up to December 15, 2022. An extensive search of the PubMed, Scopus, Cochrane, and Embase databases was performed, focusing on the effects of SARS-CoV-2 on male and female reproductive function, including testicular function, ovarian function, sex hormones profile, and pregnancy outcomes. To accomplish this, the following key strings were used: TITLE-ABS-KEY (“COVID19” OR “COVID-19” OR “SARS-CoV-2” OR “COVID” OR “SARS-CoV” OR “coronavirus” OR “SARS” OR “SARS-CoV”) AND TITLE-ABS-KEY (“Sertoli” OR “Leydig” OR “testicle” OR “testis” OR “spermatozoa” OR “sperm” OR “spermatogenesis” OR “fertility” OR “testosterone” OR “FSH” OR “LH” OR “hypogonadism” OR “ovary” OR “estradiol” OR “ovulation” OR “granulosa” OR “oocyte” OR “pregnancy” OR “ART” OR “assisted reproductive tech*” OR “IVF” OR “in vitro fertil*” OR “ICSI” OR “intracytoplasmic sperm injection” OR “IUI” OR “intrauterine insemination” OR “miscarriage” OR “LBR” OR “live birth rate”) AND (LIMIT-TO (DOCTYPE, “ar”)) AND (LIMIT-TO (SUBJAREA, “MEDI”)) AND (LIMIT-TO (LANGUAGE, “English”)). Additional manual searches were conducted using the reference lists of the relevant studies. The search was limited to English language studies.

### Selection criteria

Articles were assessed for eligibility using the Population, Exposure, Comparison/Comparator, Outcome, and Study type (PECOS) model system [39]. Specifically, we included articles on men with active or anamnestic SARS-CoV-2 infection comparing their testicular function (conventional sperm parameters and sex hormones) with that of uninfected men. Unhealthy men suffering from urogenital infections, which would have influenced the results, were excluded. Articles documenting the presence of SARS-CoV-2 RNA in the seminal fluid of infected patients were also included. Original human studies were included, while animal studies, case reports, and non-original studies such as reviews or comments were excluded from the analysis (Table 1).

**Table 1:** Inclusion and exclusion criteria according to the PECOS model [30]

	Inclusion criteria	Exclusion criteria
Population	Male participants	Pre-existing andrological disease, orchitis, azoospermia
Exposure	Active or anamnestic SARS-CoV-2 infection	–
Comparison	No infection	–
Outcomes	• Sperm conventional parameters • FSH, LH, total testosterone, prolactin, E_2_, SHBG • SARS-CoV-2 RNA in the semen fluid	WHO 5th manual not used for semen analysis
Study type	Observational studies, randomized controlled studies, case-control studies	Animal studies, in vitro studies, review and meta-analyses, case reports, book chapters, editorials, commentaries

*E*_*2*_ 17β-estradiol, *FSH* follicle-stimulating hormone, *LH* luteinizing hormone, *SARS-CoV-2* severe acute respiratory syndrome coronavirus 2, *SHBG* sex hormone-binding globulin

Articles selection was performed independently by two authors (M.M. and R.C). The titles and abstracts of the studies were first screened independently for inclusion. The decisions were reviewed by two reviewers unblinded (R.A.C. and S.L.V.). Disagreements between the authors were resolved by reviewers, while disagreements between the reviewers were resolved by a senior author (A.E.C.). Finally, the eligible articles underwent data extraction.

### Data extraction

Information was collected on the first author, year of publication, study design, duration of infection (if available), age, body mass index (BMI), LH, follicle-stimulating hormone (FSH), TT, prolactin, 17β-estradiol (E_2_), sex hormone-binding globulin (SHBG), sperm concentration, total sperm count, progressive and total motility, SARS-CoV-2 mRNA in semen in cases and controls. These parameters were collected even if reported stratified according to the severity of the COVID-19 infection. When a value was available in a different unit of measure, it was converted according to the conversion tables. For each parameter, the number of people (COVID positive/COVID negative), mean value, standard deviation (SD), median value, and interquartile range (IQR) range were reported. For the studies expressing data as median and IQR, the formula by Wan et al. [40] was used to estimate the mean and SD. Data were independently extracted by two authors. Differences between reviewers were discussed until a consensus was reached.

### Quality assessment

The quality of evidence (QoE) of the included studies was assessed by two researchers (M.M. and R.C.) using the Cambridge Quality Checklists [41]. Any disagreement between the two investigators was resolved through discussion with the other two researchers (R.A.C. and S.L.V.).

The Cambridge Quality Checklists [41] consists of three checklists to identify high-quality studies of (1) correlates, (2) risk factors, and (3) causal risk factors. The correlates checklist evaluates the appropriateness of the sample size and the quality of the outcome measurements. The risk factor checklist assigns high-quality scores to those studies with appropriate time-ordered data. Finally, the causal risk factors checklist assesses the type of study design. To draw confident conclusions about correlates, risk factors, and causal risk factors, all three checklist scores must score high.

### Statistical analysis

Quantitative data analysis was performed using Comprehensive Meta-Analysis Software (Version 3) (Biostat Inc., Englewood, NJ, USA). Standardized mean difference (SMD) has been calculated for statistical comparison between cases and controls. Statistical significance was accepted for *p* values less than or equal to 0.05. The Cochran-*Q* and heterogeneity indexes (*I*^2^) were used to assess statistical heterogeneity. In particular, if *I*^2^ was less than or equal to 50%, the variation of the studies was considered homogenous and the fixed effect model was adopted to calculate the pooled effect size. However, if *I*^2^ was greater than 50%, there was significant heterogeneity between studies, and the random effects model was used. Publication bias was qualitatively analyzed by the asymmetry of the funnel plot, which suggested some missing studies on one side of the graph. For quantitative analysis of publication bias, we used Egger’s intercept test, which assessed the statistical significance of publication bias. In this occurrence, unbiased estimates were calculated using the “trim and fill” method.

## Results

**Fig. 1:**
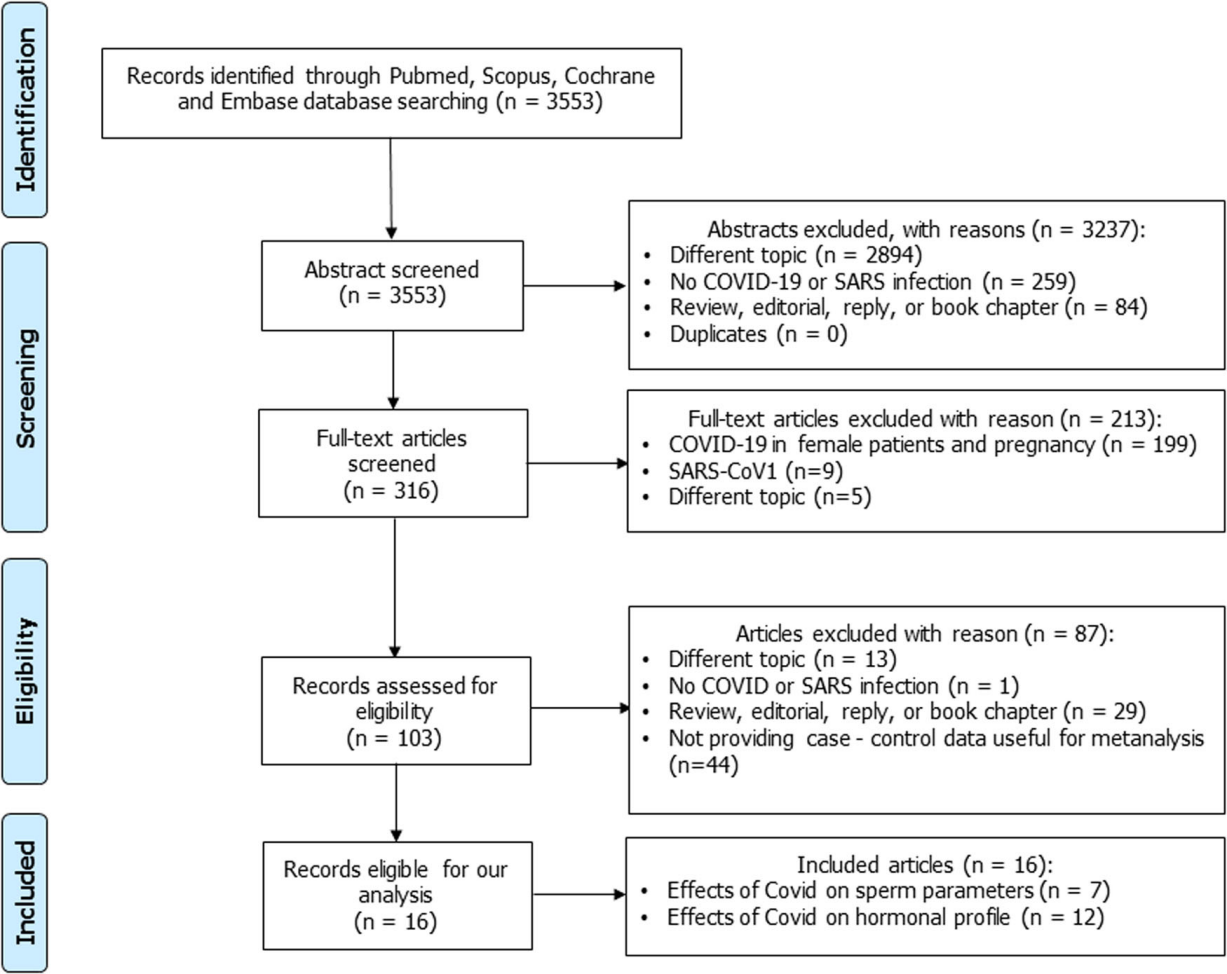
PRISMA flow diagram of literature screening

Using the aforementioned search strategy, we found 3553 articles that were screened by title and abstract. Of these, 3237 were judged not pertinent to reading their abstracts, for the following reasons: 2894 reported a different topic, 259 did not report information about SARS-Cov-2 infection, and 84 were excluded as they were reviews, editorials, case reports, or book chapters. The remaining 316 articles were evaluated for eligibility based on the reading of their full text. Of these, 300 were excluded, with reasons (Fig. 1). The remaining, 16 articles met our inclusion criteria. They included a total of 1250 patients with an active or anamnestic COVID-19 infection and 1232 age- and BMI-matched healthy controls with no prior diagnosis of urological or andrological disease. The articles included in our study were divided into two different groups: a group of 7 articles [42–49] evaluated the effect of SARS-CoV-2 infection on sperm parameters in cases and controls and a group of 12 cohort articles [20, 21, 23, 44, 49–56] evaluated the impact of SARS-CoV-2 on one or more serum hormones (LH, FSH, TT, prolactin, E_2_, and SHBG). Of these, 7 assessed LH and FSH [20, 21, 23, 44, 49–51, 55], 8 assessed TT [20, 23, 42, 49–51, 53–55, 57], 4 assessed E_2_ levels [42, 50, 54, 55], 3 analyzed prolactin levels [42, 44, 50], and 2 reported the values of SHBG [53, 54]. The main characteristic of the studies selected for meta-analysis is described in Table 2.

**Table 2:** Characteristics of the 16 studies included in the meta-analysis

Author, year	Type of study	Number of patients (case/control)	Age (median ± SD) (case/control)	BMI (median ± SD) (case/control)	Diagnosis	Clinical course/Severity of infections	Time point of semen/sex hormones testing (days from infection)	Symptoms at testing time	Duration of infection	Confonders (smoke, DM, drugs)	Main conclusion
Cinislioglu et al. [20]	Prospective cohort study	358/92	64.9 ± 11.6/67.2 ± 13.6	25.9 ± 3.8/26.4 ± 3.1	RT-PCR positivity on nasal/pharyngeal swab	Ospedalized patients	Baseline	Pneumonia	NR	NR	Reduced TT Increased LH and FSH
Guo et al. [42]	Prospective case-control study	41/50	26/26.5	27.7 ± 0.57/ NR	RT-PCR positivity on nasal/pharyngeal swab	Post ospedalized patients, recovered from covid pneumonia	76.0 (IQR: 73.0–86.5)	NR	NR	Previous corticosteroid therapy Previous antiviral therapies	Non significative difference in sperm parameters and hormone levels between patients and healthy controls
Aboelnaga et al. [53]	Prospective case-control study	55/40	53 ± 7.3/51.8 ± 7.5	35 ± 5.8/34 ± 6	NR	Post ospedalized patients, recovered from covid pneumonia	70	Pneumonia	NR	Smoke, obesity	Reduced TT in patients
Camici et al. [54]	Case-control cross-sectional study	24/24	51/50	NR	RT-PCR positivity on nasal/pharyngeal swab	Ospedalized patients	9	Pneumonia	NR	Obesity, DM, COPD in 58%	Reduced TT in patients
Salonia et al. [55]	Case-control cross-sectional study	286/281	57.6 ± 12.6/44.3 ± 12.67	27.9 ± 4.3/25.4 ± 3	RT-PCR positivity on nasal/pharyngeal swab	1.Patients discharged home 2.Patients in internal medicine 3.Patients in ICU successfully extubated 4.Patients in ICU who eventually died	Baseline	Pneumonia	NR	Arterial hypertension in 39%	85% secondary hypogonadism 9% primary hypogonadism 1% compensated hypogonadism
Temiz et al. [44]	Case-control, prospective study	10/10	38 ± 8/36.6 ± 9.6	25.5 ± 2/26.5 ± 2.7	RT-PCR positivity on nasal/pharyngeal swab	Hospitalized patients in protocol treatment with hydroxychloroquine and heparin	Baseline	Pneumonia	NR	Smoking in 30% Arterial hypertension in 5%	Reduced sperm morphology Reduced FSH, LH, TT
Kadihasanoglu, [21]	Case-control, prospective study	89/143	49.9/50	NR	RT-PCR positivity on nasal/pharyngeal swab	Hospitalized patients	Baseline	Pneumonia	NR	NR	Reduced TT Increased LH and FSH
Okçelik et al. [23]	Case-control, prospective study	24/20	35.5 ± 10.25	NR	RT-PCR positivity on nasal/pharyngeal swab or COVID pneumonia documented on CT scan	Outpatients	Baseline	Pneumonia CT documented	NR	NR	Increased LH in patients positive to RT-PCR TT reduced in patients with Covid Pneumonia
Schroeder et al. [59]	Cohort retrospective study	39/27	61.3 ± 3.84/66.6/13.3	28 ± 5/25 ± 3	RT-PCR positivity on nasal/pharyngeal swab	ICU patients	Baseline at admission in ICU	Pneumonia	NR	Obesity 28% Arterial hypertension 51% Coronary artery disease 21% DMII 33%	Reduced TT Increased estradiol
Best et al. [45]	Case-control, prospective study	30/30	–	26.2 ± 3.5/NR	RT-PCR positivity on nasal/pharyngeal swab	Outpatient with previous Covid-19 infection	37 days	Recovered	NR	NR	Lower sperm concentration Lower total sperm number
Xu et al. [50]	Case-control, cross-sectional study	39/21	60/62	25.1 ± 2.8/26.9 ± 3.6	RT-PCR positivity on nasal/pharyngeal swab OR onset of symptoms	Hospitalized patients	Baseline	Fever, cough	Viral shedding <50 days: 48.7% Viral shedding >50 days: 51.3%	Hypertension 41% Diabetes 15%	Non significative change in sex hormones in both groups
Ruan et al. [46]	Cohort, cross-sectional	55/145	31.2 ± 5.3/30.7 ± 4.3	24.4/NR	RT-PCR positivity on nasal/pharyngeal swab	Recovered patients	80 days (median)	Recovery state (lessened symptoms but still positive RT-PCR)	Time since recovery <90 days Time since recovery >90 days		No significative difference in hormonal profile Decline in sperm concentration, total sperm count and total motility Patients with longer recovery time had lower sperm count
Ma et al. [51]	Cohort, retrospective study	119/273	39/39	NR	RT-PCR positivity on nasal/pharyngeal swab OR serum virus antibody IgG or IgM	Recovered patients	78.5 days (median) for semen sample	Non severe patients in recover state	NR	NR	Increased LH
Li et al. [47]	Case-control, retrospective	23/22	40.8 ± 8.5/40.5 ± 5.9	NR	RT-PCR positivity on throat swab	Positive within the recent 7 days	NR	NR	NR	NR	NR
Holtmann et al. [48]	Cohort, prospective study	18/14	41.7 ± 9.6/33.4 ± 13.1	26.4 ± /3.2 24.5 ± 2.6	RT-PCR positivity on nasal/pharyngeal swab OR serum virus antibody IgG or IgM	Mild outpatients/ moderate hospitalized patients	31 days (median)	Recovered	NR	Smoke (10%)	Statistically significant impairment of sperm concentration, total number of sperm, progressive motility, total motility
Piroozmanesh, [49]	Case-control, cross-sectional study	40/40	38.2 ± 9.9/36.4 ± 13	26.2 ± 2.9/23.6 ± 2.6	RT-PCR positivity on nasal/pharyngeal swab OR serum virus antibody IgG or IgM	Active infections	NR	NR	NR	Criteria of exclusion	Increase in LH and FSH Statistically significant impairment of sperm quality (sperm concentration, sperm motility, and normal sperm morphology)

*CI* confidence interval, *COPD* chronic obstructive pulmonary disease, *CT* computerize tomography, *DM* diabetes mellitus, *FSH* follicle-stimulating hormone, *ICU* intensive care unit, *IQR* interquartile range, *LH* luteinizing hormone, *NR* not reported, *RT-PCR* real-time polymerase chain reaction, *SD* standard deviation, *TT* total testosterone

### Quality of evidence of included studies

The quality of evidence (QoE) of the studies was assessed by 2 investigators (RC and MM), using the Cambridge Quality Checklist [41]. Although this scale does not establish a precise threshold for differentiating between high and low-quality studies, out of a total score of 15, 1 study scored >10, the remaining studies scored 6 to 10. No study achieved a score lower than 6 (Table 3).

**Table 3:** Quality of evidence of the included studies according the Cambridge Quality Checklists

Authors	Type of study	Checklist for correlates	Checklist for risk factor	Checklist for causal risk factors	Total
Cinislioglu et al. [43]	Prospective cohort study	2	3	4	9
Guo et al. [33]	Prospective case-control study	3	3	4	10
Aboelnaga et al. [45]	Prospective case-control study	3	3	4	10
Camici et al. [46]	Case-control cross-sectional study	3	1	5	9
Salonia et al. [47]	Case-control cross-sectional study	3	1	6	10
Temiz et al. [35]	Case-control, prospective study	3	3	4	10
Kadihasanoglu [49]	Case-control, prospective study	3	3	4	10
Okçelik et al. [50]	Case-control, prospective study	3	3	4	10
Schroeder et al. [52]	Cohort retrospective study	4	2	6	12
Best et al. [36]	Case-control, prospective study	3	2	4	9
Xu et al. [41]	Case-control, cross-sectional study	3	2	4	9
Ruan et al. [37]	Cohort, cross-sectional	4	1	4	9
Ma et al. [42]	Cohort, retrospective study	4	2	5	9
Li et al. [38]	Case-control, retrospective	2	2	6	10
Holtmann et al. [39]	Cohort, prospective study	3	3	4	10
Piroozmanesh. [40]	Case-control, cross-sectional study	2	3	4	9

#### Age in patients vs. controls

Pooled analysis of the 16 studies revealed that age was not significantly different between patients and controls (SMD 0.211; CI 95% −0.205, 0.628; *p* = 0.320) (Supplementary Fig. 1). The analysis showed the presence of heterogeneity between studies as shown by the *I*^2^ test (*I*^2^ = 91%, *p* = 0.000) and the *Q*-test (*Q*-value = 102.594). We found the absence of publication bias, as shown by the symmetry of the funnel plots (Supplementary Fig. 2A), and the result of Egger’s test (intercept −2.39430, 95% CI −7.27079, 2.48249; *p* = 0.14517). Any study was found sensitive enough to alter the results (Supplementary Fig. 2B).

#### BMI in patients vs. controls

Seven studies [20, 48–50, 53, 58, 59] reported information on BMI for patients and controls. BMI was not significantly different (SMD 0.341; CI 95% −0.043, 0.724; *p* = 0.082) (Supplementary Fig. 3). The analysis showed heterogeneity between studies (*I*^2^ = 87%, *p* = 0.000; *Q* = 55.314) and absence of publication bias, as derived from the symmetry of the funnel plots (Supplementary Fig. 4A) and Egger’s test result (intercept −0.59774, 95% CI −5.71742, 4.52194; *p* = 0.39236). Two studies were sensitive enough to alter the results [20, 50] (Supplementary Fig. 4B).

#### LH in patients vs. controls

**Fig. 2:**
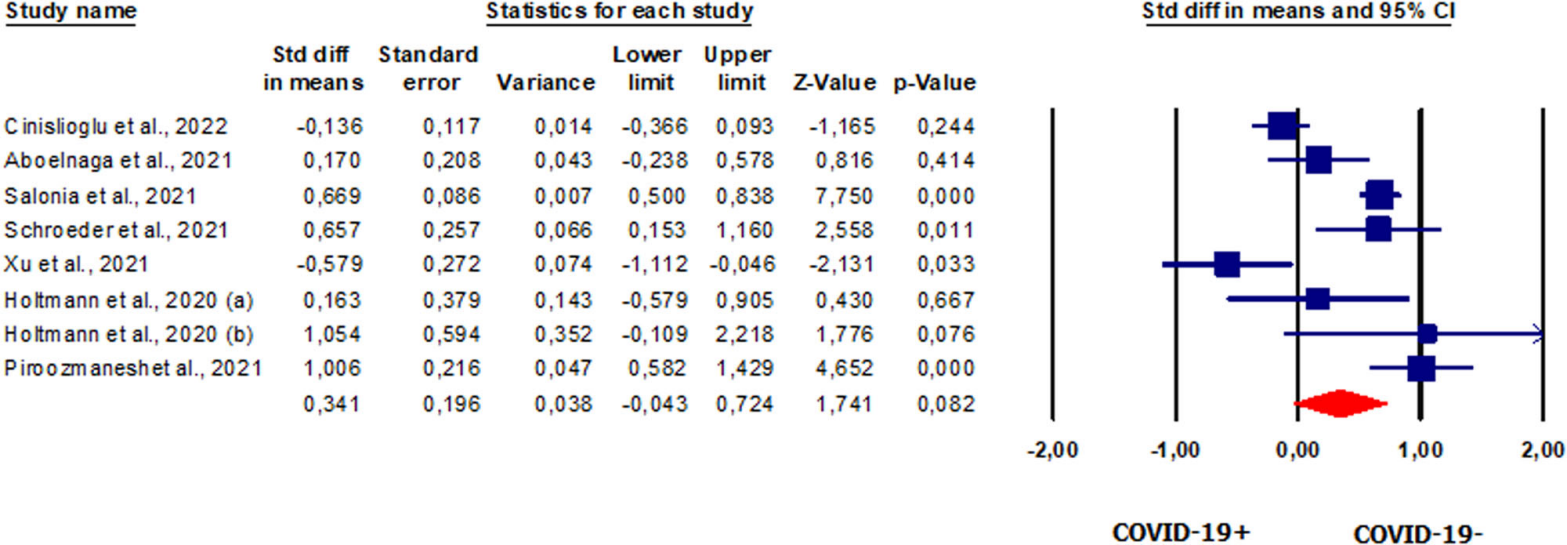
Forest plot of the luteinizing hormone in patients and controls

Seven studies [20, 23, 42, 49, 51, 53, 55] including 824 patients assessed serum LH levels. Our analysis showed that LH levels were significantly higher in patients than in controls (SMD 1.229; CI 95% 0.372, 2.087; *p* = 0.005) (Fig. 2). The analysis showed the presence of heterogeneity between studies, as derived by the *I*^2^ test (*I*^2^ = 97%, *p* = 0.000), and the *Q*-test (*Q* = 278.960). The analysis showed the absence of publication bias as inferred by Egger’s test (intercept 6.91812, 95% CI −4.80247, 18.63892; *p* = 0.094) and the symmetry of the funnel plots (Supplementary Fig. 5A). No study was sensitive enough to alter the results (Supplementary Fig. 5B).

#### FSH in patients vs. controls

**Fig. 3:**
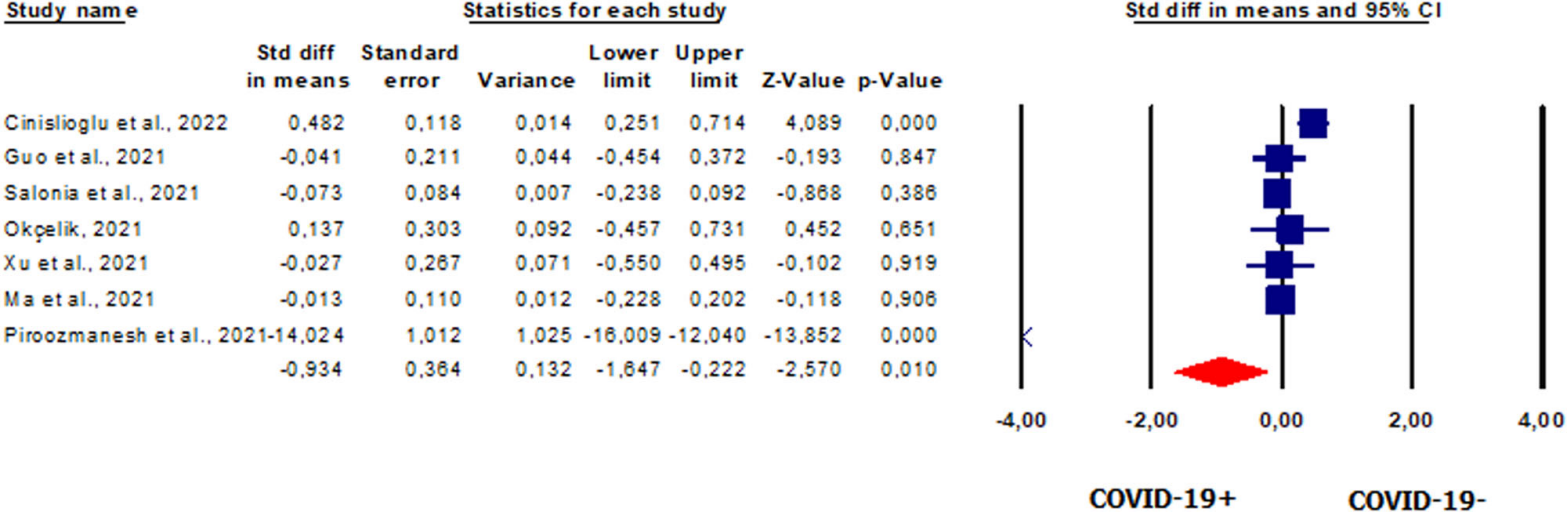
Forest plot of the follicle-stimulating hormone in patients and controls

Seven studies [20, 23, 42, 49–51, 58], with a total of 808, reported serum FSH levels. Pooled analysis showed that FSH levels were significantly lower in patients vs. controls (SMD −0.934, CI 95% −1.647, −0.222, *p* = 0.010) (Fig. 3). The analysis showed the presence of heterogeneity between studies as shown by the *I*^2^ test (*I*^2^ = 97%, *p* = 0.000) and the *Q*-test (*Q* = 209.335). The analysis showed the absence of publication bias, as shown by the funnel plots and Egger’s test results (intercept 6.477846, 95% CI −16.30546, 3.34855; *p* = 0.07546) (Supplementary Fig. 6A). One study was sensitive enough to alter the results [49] (Supplementary Fig. 6B).

#### Total testosterone in patients vs. controls

**Fig. 4:**
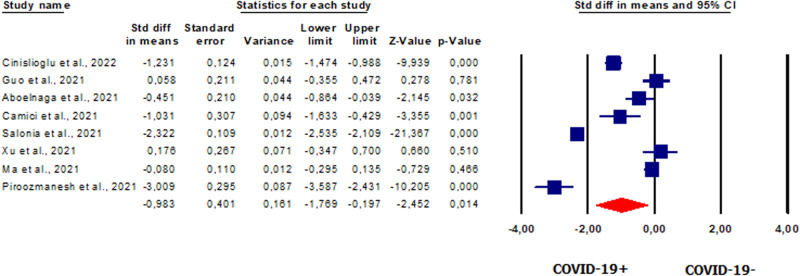
Forest plot of total testosterone in patients and controls

We conducted an overall analysis of serum TT levels expressed in nmol/l. Eight studies [20, 42, 49–51, 53, 54, 58] reported TT levels in a total of 887 patients. Our analysis showed that TT levels were significantly lower in patients than in controls (SMD −0.983, CI 95% −1.769, −0.0197, *p* = 0.014) (Fig. 4). *I*^2^ and *Q* tests showed the presence of heterogeneity between studies (*I*^2^ = 97.8%, *p* = 0.000; *Q* = 317.934). The analysis showed the absence of publication bias, as shown by the symmetry for the funnel plots (Supplementary Fig. 7A) and Egger’s test (intercept 2.23248, 95% CI −14.00580, 18.46877; *p* = 0.37406). One study was sensitive enough to alter the results [49] (Supplementary Fig. 7B).

#### Prolactin in patients vs. controls

**Fig. 5:**
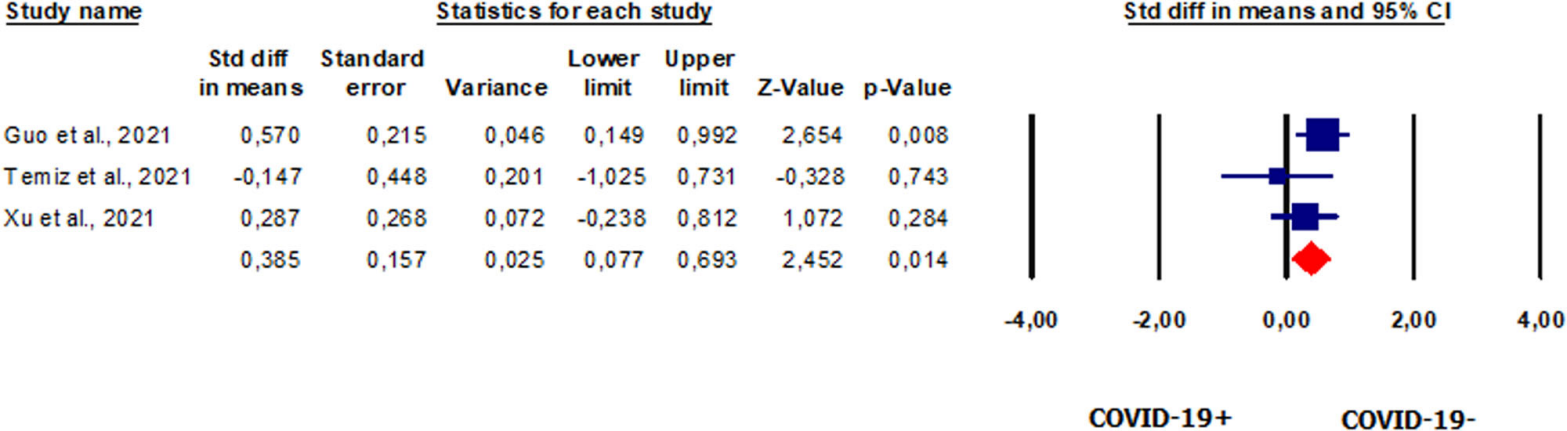
Forest plot of prolactin in patients and controls

We conducted our analysis on three studies that reported serum prolactin levels in a total of 90 patients [44, 50, 52]. We found that the levels of this hormone were significantly higher in patients than in healthy controls (SMD 0.385, CI 95% 0.077, 0.693, *p* = 0.014) (Fig. 5). The analysis showed no heterogeneity between the studies (*I*^2^ = 12.6%, *p* = 0.32; *Q*-test = 2.289). No publication bias was revealed in the analysis, as shown by the symmetry of the funnel plots (Supplementary Fig. 8A) and Egger’s test (intercept −3.12696, 95% CI −12.92849, 6.67458; *p* = 0.07699). The sensitivity analysis revealed one study [42] that could alter the results (Supplementary Fig. 8B).

#### 17ß-estradiol in patients vs. controls

**Fig. 6:**
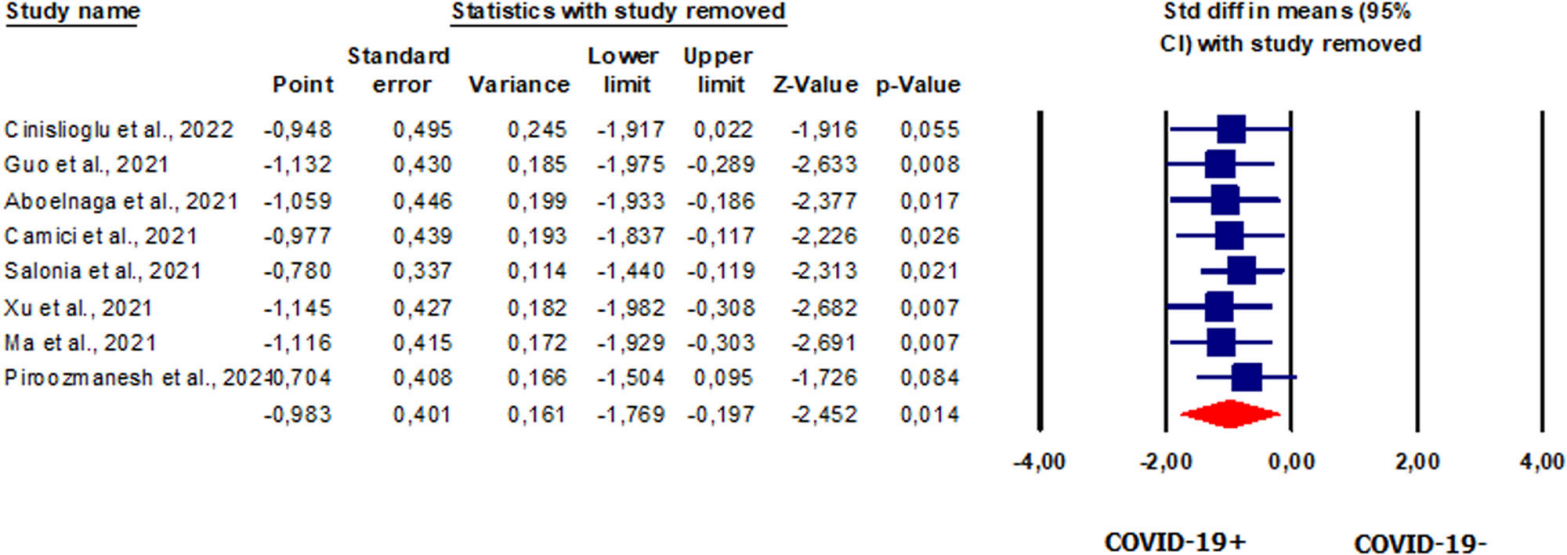
Forest plot of 17β-estradiol in patients and controls

We found four studies [42, 50, 54, 58] that evaluated serum E_2_ levels in a total of 390 patients. The values were reported in pg/ml. Our analysis found that E_2_ levels were significantly higher in patients than in healthy controls (SMD 0.524, CI 95% 0.113, 0.935, *p* = 0.012) (Fig. 6). The *I*^2^ test showed heterogeneity between studies (*I*^2^ = 76.8%, *p* = 0.005; *Q* = 12.932). The analysis showed the absence of publication bias, as shown by the symmetry of the funnel plot (Supplementary Fig. 9A) and the results of Egger’s test (intercept –2.67325, 95% CI −10.26546, 4.91896; *p* = 0.13450). Two studies were sensitive enough to alter the results [50, 55] (Supplementary Fig. 9B). In the study of Xu et al. [50], as explained by the authors, this could be related to the E_2_ measurement technique, which was done by radioimmunoassays. The authors suggest that it has lower precision and sensitivity than the new analysis techniques and may cause measurement errors.

#### SHBG in patients vs. controls

**Fig. 7:**
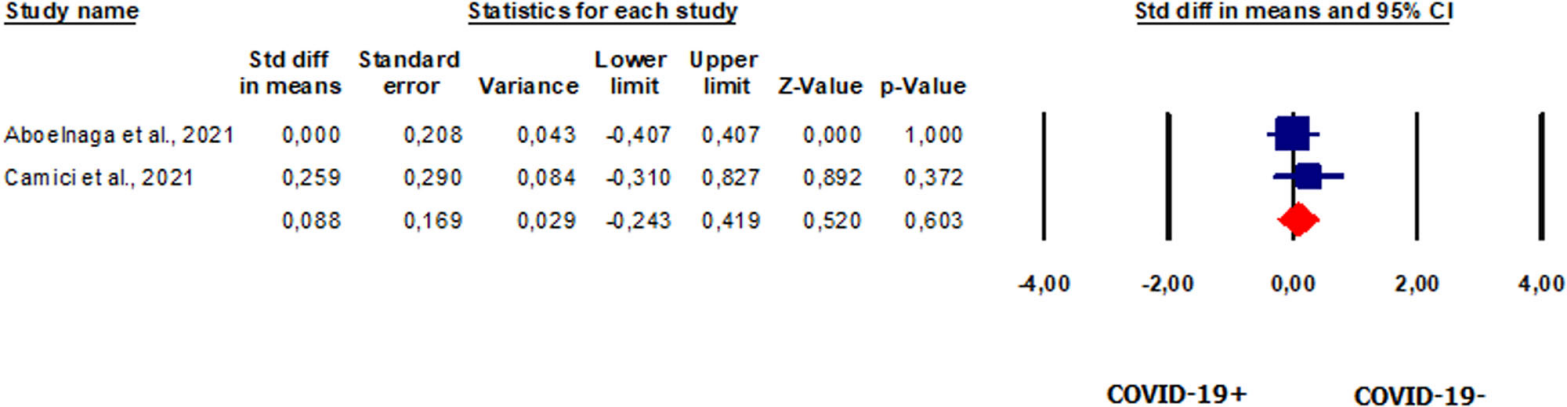
Forest plot of sex hormone-binding globulin in patients and controls

We evaluated two studies [53, 54] that reported SHBG values in 79 COVID-19 patients. The analysis showed that serum SHBG levels were not significantly different in patients compared to controls (SMD 0.088, CI 95% −0.243, 0.419, *p* = 0.603) (Fig. 7). The analysis showed no heterogeneity between the studies (*I*^2^ = 0%, *p* = 0.47, *Q* = 0.526). Analysis of publication bias could not be performed due to the limited number of studies. Neither study was found sensitive enough to alter the results (Supplementary Fig. 10).

#### Sperm concentration in patients vs. controls

**Fig. 8:**
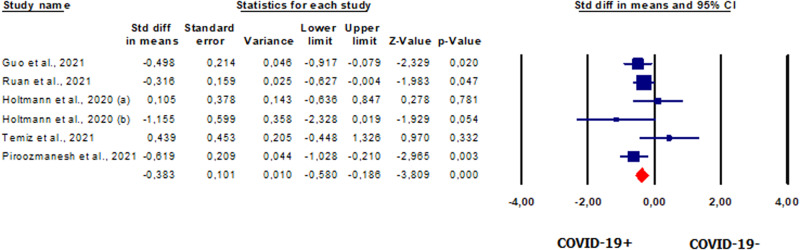
Forest plot of sperm concentration in patients and controls

Five studies [42, 44, 46, 48, 49] involving 207 patients evaluated sperm concentration. Our analysis revealed that sperm concentration was significantly lower in patients than in healthy controls (SMD −0.383, CI 95% −0.58, −0.186, *p* = 0.000) (Fig. 8). The analysis showed no heterogeneity between the studies (*I*^2^ = 40%, *p* = 0.13, *Q* = 8.373). Also, the symmetry of the funnel plots (Supplementary Fig. 11A) and the results of Egger’s test (intercept 0.57986, 95% CI −3.38352, 4.54323; *p* = 0.35269) showed the absence of publication bias. No study was sensitive enough to alter the results (Supplementary Fig. 11B).

#### Total sperm count in patients vs. controls

**Fig. 9:**
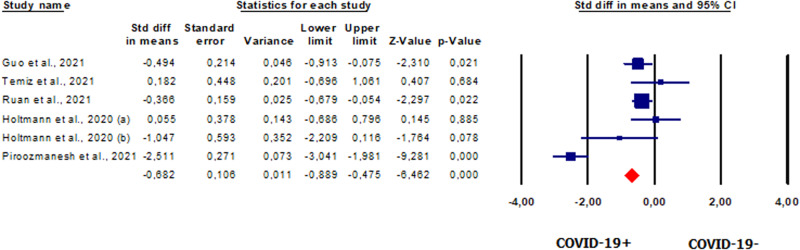
Forest plot of total sperm count in patients and controls

Five studies [42, 44, 46, 48, 49] involving 214 patients evaluated total sperm count. The analysis showed significantly lower total sperm count in patients than in healthy controls (SMD −0.682, CI 95% −0.889, −0.475, *p* = 0.000) (Fig. 9). Heterogeneity was found between studies (*I*^2^ = 91%, *p* = 0.000, *Q* = 58.291). The analysis showed the absence of publication bias, as shown by the symmetry of the funnel plot (Supplementary Fig. 12A) and Egger’s test (intercept −0.63931, 95% CI −11.27831, 9.99969; *p* = 0.43780). No study was sensitive enough to alter the results (Supplementary Fig. 12B).

#### Progressive motility in patients vs. controls

**Fig. 10:**
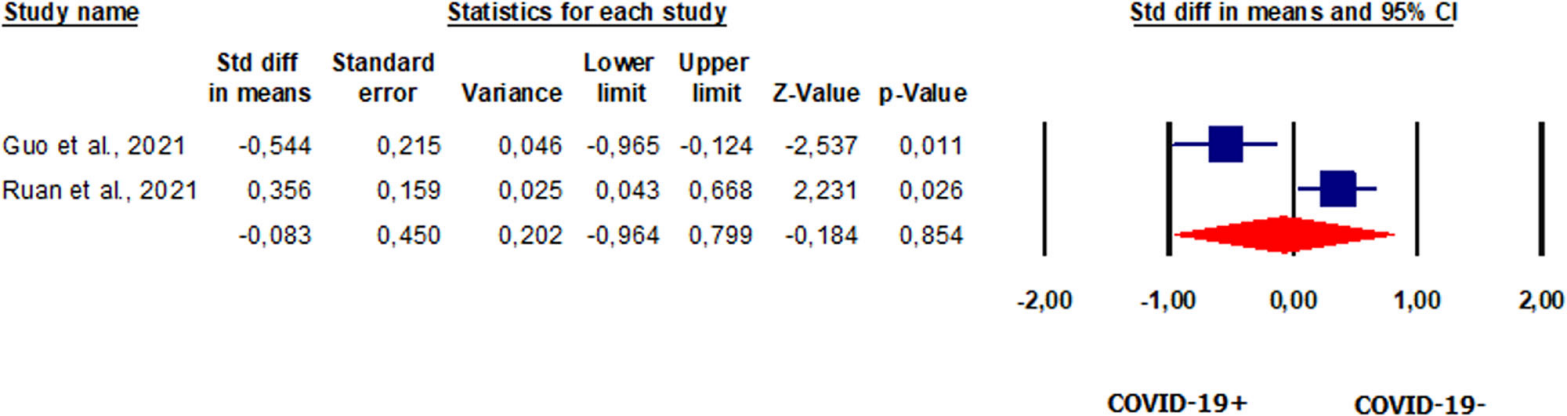
Forest plot of progressive sperm motility in patients and controls

Two studies [42, 46] evaluated progressive sperm motility, which was not significantly different in the 96 patients enrolled in both studies compared to healthy controls (SMD −0.083, CI 95% −0.964, 0.799, *p* = 0.854) (Fig. 10). *I*^2^ and the *Q* tests showed the presence of heterogeneity between studies (*I*^2^ = 91%, *p* = 0.001, *Q* = 11.335). Analysis of publication bias could not be performed due to the limited number of studies. Sensitivity analysis revealed that both studies were sensitive enough to alter the results (Supplementary Fig. 13). Interestingly, Guo et al. [42], in the subgroup analysis of men who provided a second semen sample, obtained 80 days (IQR 74.0–89.0) after hospital discharge, found no significant improvement in progressive motility.

#### Total motility in patients vs. controls

**Fig. 11:**
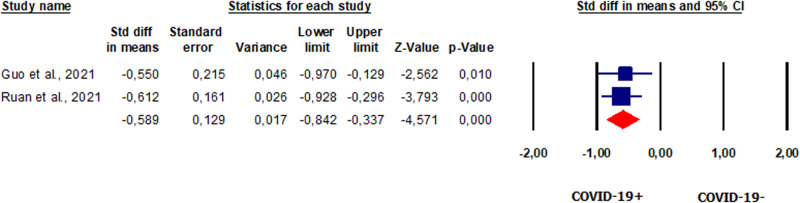
Forest plot of total sperm motility in patients and controls

Total sperm motility was evaluated in two studies [42, 46] involving 96 patients. Our analysis showed that total motility was significantly lower in patients than in controls (SMD −0.589, CI 95% −0.842, −0.337, *p* = 0.000) (Fig. 11). The analysis showed no heterogeneity between studies (*I*^2^ = 0%, *p* = 0.817, *Q* = 0.053). Analysis of publication bias could not be performed due to the limited number of studies. Sensitivity analysis found that these studies were not sensitive enough to alter the analysis (Supplementary Fig. 14).

#### SARS-CoV-2 mRNA detection

Seven studies [44–49, 60] evaluated the presence of SARS-CoV-2 mRNA in the semen of patients and controls. None of them found viral mRNA in the semen.

## Discussion

### Background literature

Since the COVID-19 outbreak, many studies have been conducted to evaluate the consequences of acute SARS-CoV-2 infection. These mainly concern respiratory function and the disease-related severe inflammation, resulting in an acute multidistrict inflammatory reaction, but many human apparatuses and systems are also affected by the virus. Thus, research interest has mostly focused on the prevention and management of life-threatening conditions, while little is known about the effects of COVID-19 on the endocrine and genital systems. While long-term sequelae of virus exposure are not yet clearly detectable, an increased prevalence of orchitis and epididymitis has been reported in the pandemic years, suggesting the involvement of the virus in acute male urogenital tract infection [8, 61]. This has been the subject of cohort studies, case reports, and trials [8, 47, 61–64]. Retrospective and prospective studies have also been conducted to evaluate the influence of the virus on sperm parameters and male sex hormones [47, 64].

In this regard, we reviewed data from four recently published meta-analyses. Of these, two investigated the effects of the virus both on sex steroid hormones and sperm parameters [65, 66], while the other two only reported data from studies on semen analysis [67, 68].

Specifically, the studies reported LH, FSH, TT, semen volume, sperm concentration, total sperm count, total, non-progressive, and progressive sperm motility, and sperm morphology. Only one meta-analysis [65] included studies reporting E_2_ and prolactin levels, while none of them considered SHBG values. The results of these meta-analyses are summarized in Table 4.

**Table 4:** Results of previous meta-analyses published in 2022

Authors	Corona et al. [60]	Xie et al. [61]	Wang et al. [59]	Chen et al. [62]
Patients/Controls	2092/1138	213/289	1627/1535	521/653
Time of hormonal sampling since RT-PCR positivity	Acute phase of disease	NE	>90 days	NE
LH	No significant difference—CI 95% 1.11 (−0.20, 2.42)	NE	No significant difference	NE
FSH	No significant difference—CI 95% 0.39 (−0.70, 1.39)	NE	No significant difference	NE
TT	Significantly reduced—CI 95% −3.95 (−6.73, −1.17)	NE	No significant difference	NE
E_2_	NE	NE	Significantly increased—CI 95% 0.65 (0.25, 1.05)	NE
PRL	NE	NE	Significantly increased—CI 95% 0.30 (0.045, 0.57)	NE
SHBG	NE	NE	NE	NE
Semen collection time since RT-PCR positivity	Acute phase of disease	14–40 days	<90 days	90 days
Semen volume	Significantly reduced—CI 95% −0.94 (−1.73, −0.14)	Significantly reduced—CI 95% −0.17 (−0.36, 0.01)	No significant difference	No significant difference—CI 95% −0.49 (−1.03–0.05)
Sperm concentration	Significantly reduced—CI 95% −28.25 (−46.31, −10.19)	Significantly reduced—CI 95% −0.37 (−0.78, 0.03)	Significantly reduced—CI 95% −0.42 (−0.65, −0.2)	No significant difference—CI 95% −11.24 (−29.93, 7.45)
Total sperm count	Significantly reduced—CI 95% −95.86 (−18.071, −11.01)	Significantly reduced—CI 95% −0.35 (−0.57, −0.12)	Significantly reduced—CI 95% −0.4 (−0.7, −0.09)	No significant difference—CI 95% −61.33 (−128.46, 5.80)
Progressive sperm motility	No significant difference (analysis not shown)	Significantly reduced—CI 95% −0.13 (−0.64, 0.37)	Significantly reduced—CI 95% −0.3 (−0.7, −0.003)	No significant difference—CI 95% −5.98 (−16.03, 4.07)
Non-progressive sperm motility	NE	NE	No significant difference	NE
Total sperm motility	Significantly reduced—CI 95% −7.52 (−11.10, −3.94)	Significantly reduced—CI 95% −0.57 (−0.88, −0.25)	Significantly reduced—CI 95% −0.6 (−0.96, −0.2)	Significantly reduced—CI 95% −5.37 (−8.47, −2.28)
Normal sperm morphology	No significant difference (analysis not shown)	NE	No significant difference (analysis not shown)	No significant difference—CI 95% −4.05 (−9.72, 1.61)

*CI* confidence interval, *E*_*2*_ 17β-estradiol, *FSH* follicle-stimulating hormone, *LH* luteinizing hormone, *NE* not evaluated, *PRL* prolactin, *SHBG* sex hormone-binding globulin, *TT* total testosterone

The finding of low serum TT in patients with current or previous COVID-19 infection achieved statistical significance in both meta-analyses evaluating the effect of the virus on sex hormones [65, 66]. These data are in agreement with the results of our study. In contrast to our data, which reported increased LH levels in infected patients, a non-significant difference in LH and FSH levels was reported in both meta-analyses. The only meta-analysis that investigated E_2_ and prolactin levels reported a statistically significant increase in both hormones in patients compared with controls [65], consistent with our results. A significant reduction in semen volume was reported in two meta-analyses [66, 67], while the other two did not report a statistically significant difference. Sperm concentration, and total sperm count were significantly reduced in infected patients in the meta-analyses by Corona et al. [66], Xie et al. [67], and Wang et al. [65], while Chen et al. [68] found no significant differences between the two groups. Progressive sperm motility has been reported to be reduced in patients compared to controls by Xie et al. [67] and Wang et al. [65], but not by Corona et al. [66] and Chen et al. [68]. These results are in line with ours, except for progressive motility for which we did not find a statistically significant difference. Finally, only one meta-analysis [65] evaluated the effects of infection on non-progressive motility which was not statistically different between patients and controls. Total motility was significantly affected in all analyses, as also reported in the present study. Finally, the studies evaluating sperm morphology showed no statistically significant effect on this parameter by SARS-CoV-2 infection. Concerning the follow-up time, these meta-analyses mainly included patients with active or recent infection, while it is unknown whether these results would be confirmed after longer follow-up times. Corona et al. [66] conducted their study on hormonal profile and semen samples collected from patients in the acute phase of the disease. Xie et al. [67] guided the study on semen samples from an average of 14–40 days after positivity for the virus. Wang et al. [65] data on the hormonal study were performed >90 days from positivity, therefore in a recovery phase. The authors themselves conclude that a longer follow-up would be necessary to evaluate the consequences of COVID on male hormone profile. In the same study, semen samples were instead collected at a median time ≤90 days after infection, in the acute phase of viral infection, to assess the consequences of the virus on male gametes. Similarly, Chen et al. [68] conducted their study on semen sampled 90 days after infection.

### Our findings

In line with previous evidence, we analyzed the data of a short follow-up time in keeping with most of the data published so far. Indeed, hormonal evaluation and sperm analysis were performed in a period ranging from baseline (acute infection) up to 80 days from infection. We found a decrease in TT levels in male patients with active or anamnestic COVID-19 infection, thus a condition of hypotestosteronemia that could alter the response of male patients to the infection and compromise spermatogenesis. This alteration suggests the direct role of COVID-19 on testicular function, also supported by the finding of an alteration of spermatogenesis. The mechanisms that cause hypotestosteronemia may relate to inflammation. Indeed, SARS-CoV-2 infection causes inflammation and increases oxidative stress [69, 70], which leads to reduced androgen synthesis and, in parallel, generates increased E_2_ biosynthesis due to increased aromatase activity [71]. The increased activity of this enzyme could be due to obesity. The latter is a known risk factor for severe COVID-19 [72] and shares with this disease some common mechanisms, such as immune system activity attenuation and chronic inflammation [73]. Furthermore, obesity, and, in particular visceral obesity, is a well-known risk factor for testosterone deficiency in men [74–76]. However, we found no differences in BMI between patients and controls. This observation makes a role of obesity in the pathogenesis of hypotestosteronemia unlikely, while it suggests a role of SARS-CoV2 infection.

Our analysis confirms other data in the literature showing that low testosterone levels are associated with high LH levels, while FSH secretion does not appear to change significantly. Some studies, including our meta-analysis, have also shown that prolactin is significantly higher in patients with COVID-19 than in healthy men [77]. This could be due to the stress of being unwell and/or the medications being administered, but the exact mechanism and long-term consequences of elevated prolactin levels are still unclear. Hyperprolactinemia has been suggested to play a role in the pathophysiology of the hypogonadism observed in male patients with COVID-19 [78]. Furthermore, Guo et al. [42] suggested that hyperprolactinemia may be pre-existing, as no recovery was observed in a second blood draw after viral clearance; this could then alter the sensitivity analysis. Recovery time from hyperprolactinemia could provide insight into the time to the improvement of male gonadal function. Therefore, hormonal surveillance studies are needed to evaluate the influence on gonadal function and recovery time. Undoubtedly, we question a possible pathogenetic role of hyperprolactinemia in the onset of hypogonadism in patients with COVID-19 because these patients also have significantly elevated LH levels, whereas hyperprolactinemia is known to be associated with low or inappropriately low LH levels. Therefore, according to the current evidence, hypogonadism in COVID-19 patients mainly seems to be recognized as a primary mechanism. Analysis of SHBG levels did not report statistically significant differences, but its role related to testosterone and estrogen levels during inflammation and its role in COVID-19 infection has to be further investigated, as it may explain the altered levels of TT and E_2_ [79].

Finally, our analysis confirms the impairment of sperm parameters reported by other studies. Literature shows that male patients who recovered from COVID-19 infection had worse semen parameters when compared to healthy controls for COVID-19 [80]. Our short-term analysis of seminal fluid demonstrates a wide variety of semen outcomes, ranging from azoospermia to normozoospermia. We found lower sperm concentration, total sperm count, and total sperm motility. Progressive sperm motility was not significantly different, but few studies have evaluated it. These findings may reveal important concerns for male fertility, as they could reduce the fertilization rate, both in natural pregnancies and in assisted reproductive techniques. Certainly, further studies are needed as a long-term follow-up period is missing and the long-term effects of COVID-19 infection have yet to be clarified, and the recovery time of sperm parameters. The results of our meta-analysis are summarized in Table 5.

**Table 5:** Summary of the results of the present meta-analysis

Parameters	No. of studies	Patients/controls	SD in means	CI 95%	*p* value	Interpretation
LH	7	824/796	1.229	0.372, 2.087	0.005	Higher than healthy controls
FSH	7	808/778	−0.934	−1.64, −0.222	0.010	Lower that healthy controls
TT	8	887/822	−0.98	−1.769, −0.0197	0.014	Lower than healthy controls
E_2_	4	390/377	0.524	0.113, 0.935	0.012	Higher than healthy controls
PRL	3	90/82	0.385	0.077, 0.693	0.014	Higher than healthy controls
SHBG	2	79/64	0.088	−0.243, 0.419	0.603	Not significantly different
Sperm concentration	6	214/249	−0.383	−0.58, −0.186	0.000	Lower than healthy controls
Total sperm count	6	214/249	−0.682	−0.889, −0.475	0.000	Lower than healthy controls
Progressive sperm motility	2	96/195	−0.083	−0.964, 0.799	0.854	Not significantly different
Total sperm motility	2	96/195	−0.589	−0.842, −0.337	0.000	Lower than healthy controls

*CI* confidence interval, *E*_*2*_ 17β-estradiol, *FSH* follicle-stimulating hormone, *LH* luteinizing hormone, *PRL* prolactin, *SD* standard deviation, *SHBG* sex hormones-binding globulin, *TT* total testosterone

The longest follow-up study assessing semen parameters was conducted by Hu et al. [81], who conducted an additional follow-up investigation on COVID-19 patients with a median recovery time of 177.5 days compared to age-matched healthy controls for sperm quality. After a 120-day recovery period, total sperm motility did not change, and sperm count had improved, while the total number of sperm in this group was impaired. Confirmation and investigation of the precise moment at which sperm quality started to increase require bigger cohort research with varying recovery times.

Similar investigations were conducted by Ma et al. [51] who evaluated the sperm quality and sex hormone profiles in 12 male COVID-19 patients whose median period of semen collection was 78.5 days from the onset of the disease. The findings basically showed that 119 male COVID-19 patients had reduced gonadal function and some patients had lower sperm motility and increased sperm DNA fraction percentages. The study by Holtmann et al. [48] found that COVID-19 patients had significantly lower sperm quality compared to the control group when there was a mean interval of 25.5 days between the end of symptoms and semen collection. Both investigations showed that male COVID-19 patients’ sperm quality began to drop during the early stages of recovery (related to the spermatogenesis cycle). Thus, it could be hypothesized that COVID-19 patients’ sperm quality increased following a recovery period of roughly 6 months.

Concerning the detection of SARS-CoV-2 mRNA in seminal fluid, literature searches are still controversial and therefore inconclusive [82]. Most of the studies, including our meta-analysis, have not confirmed the existence of the SARS-CoV-2 virus in the semen of male COVID-19 patients, while some studies have detected the virus RNA in the semen of COVID-19-infected patients [83–85]. These latter studies suggest that the SARS-CoV-2 virus may be able to enter the testis and colonize the semen. In severe clinical cases with a high viral load and the acute phase of the disease, the systemic inflammatory response allows the virus to spread across the blood testis barrier. Consequently, the infection could impair spermatogenesis and hormone secretion in COVID-19 patients, leading to male fertility impairment. More studies need to be done as it is unclear how long it takes to clear the virus from semen. Li et al. [86] reported for the first time the detection of SARS-CoV-2 in the semen of 38 patients with a severe form of COVID-19 and during the acute phase of the disease. Therefore, the stage of the disease may be an important factor to take into account. This condition may correspond to a higher blood viral load and, therefore, a greater possibility of crossing the blood testis barrier. Interestingly, the most recent study published on this topic aimed to develop a reliable methodology capable of detecting SARS-CoV2 mRNA in the seminal fluid. The authors developed a qualitative RT-PCR assay, which was controlled for PRM1 and PRM2 mRNAs. A positive control obtained by diluting the viral preparation from a SARS-CoV-2 panel was used. Despite the precise methodology, the assay was unable to detect the presence of SARS-CoV-2 mRNA in the seminal fluid of patients with mild and recovered COVID-19 [87]. To the best of our knowledge, all studies evaluating the presence of SARS-CoV-2 in the seminal fluid are listed in Table 6. The studies included in our meta-analysis are not listed as semen analysis was negative for the SARS-CoV-2 RNA in all of them.

**Table 6:** Summary of the studies that have evaluated the presence of the SARS-CoV-2 in the seminal fluid

Article	Presence of SARS-CoV-2 mRNA in seminal fluid	Semen characteristics	Stage of the disease
Purpura et al. [83]	1 of 7	Severe oligozoospermia (<1 million/ml) Severe asthenozoospermia (0%)	Convalescence (81 days after infection)
Sharma et al. [104]	No	–	–
Saylam et al. [84]	4 of 30	NE	Severe pneumonia
Camici et al. [54]	No	–	–
Piroozmanesh et al. [49]	No	–	–
Gacci et al. [88]	1 of 43	–	Acute stage of disease, needed intensive care support
Burke et al. [105]	No	–	–
Paoli [106]	No	–	–
Temiz et al. [44]	No	–	–
Best et al. [45]	No	–	–
Ruan et al. [46]	No	–	–
Gupta [60]	No	–	–
Machado et al. [85]	1 of 15	NE	Two weeks after onset of symptoms
Li et al. [47]	No	–	–
Holtmann et al. [48]	No	–	–
Kayaaslan [107]	No	–	–
Pan et al. [100]	No	–	–

*NE* not evaluated

There is a lack of information regarding reproductive outcomes in critical and severe disease states, in contrast to mild and moderate disease states. Also unreported is the connection between disease severity, hormone levels, and semen parameters. According to Gacci et al. [88], it may be suggested a link between the severity of COVID-19 and azoospermia, with 7.0% of cases of severe oligoasthenoteratozoospermia and 18.6% of cases of azoospermia observed in severe illness. Semen parameters have been seen to revert to normal levels 79 days after the fever. Most findings suggested that 3 months following COVID-19 recovery, the overall state of andrological health appeared to be unaffected. It can be presumed that no major long-term impairment and no sperm autoimmune response had occurred during a full spermatogenetic cycle from recovery.

**Fig. 12:**
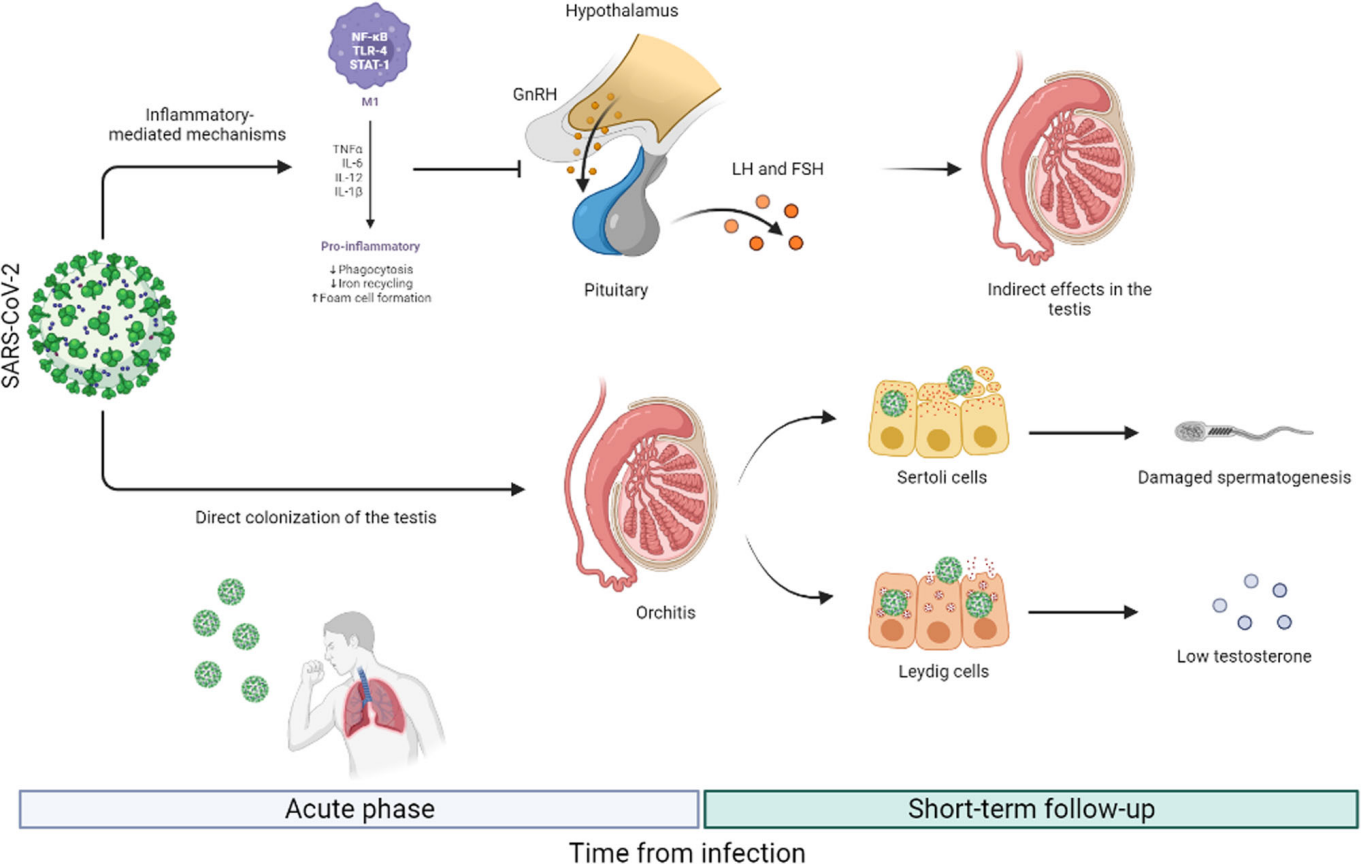
Mechanisms by which SARS-CoV-2 might damage testicular function in the acute phase and after short-term follow-up. In the acute phase, mechanisms mediated by inflammation, such as the increase in pro-inflammatory cytokines, lead to a reduction of GnRH pulses and, consequently, of serum LH and FSH levels. This is also favored by the use of glucocorticoids to treat COVID-19. After recovery, the hypothalamic-pituitary axis function will be restored. SARS-CoV-2 is also able to directly infect the testis and damage Leydig and Sertoli cells, thus leading to decreased testosterone biosynthesis and impaired spermatogenesis associated with elevated LH and unchanged FSH levels, in the short-term follow-up. The ability of these cells to regain their function will allow recovery from primary hypogonadism in the long-term follow-up. FSH follicle-stimulating hormone, GnRH gonadotropin-releasing hormone, IL interleukin, LH luteinizing hormone, SARS-CoV-2 severe acute respiratory syndrome Coronavirus 2, TNFα tumor necrosis factor α

Recent autopsy series have reported testicular pathological findings in patients with COVID-19. SARS-CoV-2 viral antigen was detected in Sertoli cells, Leydig cells, spermatids, and fibroblast cells in rete testis under electron microscopy [89]. Furthermore, viral particles were identified by immunohistochemistry in multiple testicular cells [90] and SARS-CoV2 RNA was found in testicular tissue [91]. The most frequent macroscopic findings were mild interstitial orchitis, hyperemia, interstitial edema, thickening of the basal membrane, thinning of the seminiferous tubules, increased apoptotic cells in the tubules, reduction of Leydig and Sertoli cells, and spermatogenesis impairment [91]. The microscopic series reported impaired spermatogenesis, interstitial infiltration macrophages, and leukocytes, tubular damage with swelling of Sertoli cells, vacuolation, and shedding [35, 89, 92]. This evidence confirms that the testis may be susceptible and vulnerable to COVID-19. The finding of ACE-2 and TMPRSS2 can explain the pathological aspects found in male patients who died from COVID-19 infection with the presence of SARS-CoV-2 at the testicular level. Consequently, spermatogenesis and hormone secretion in COVID-19 patients would be damaged by the virus, resulting in impaired male fertility [29] (Fig. 12). Thus, it may be suggested that in the assessment of males affected by COVID-19 and diagnosed with epididymal-orchitis, the examination of sperm parameters and hormone levels is advised [93].

### Strengths of the study

Several studies and meta-analyses have been conducted on this topic. Our study adds new perspectives to the existing literature. One of the strengths of our study is the thorough hormonal evaluation. Most studies reported only the levels of LH, FSH, and TT. A few studies included PRL, E_2_, and SHBG. These assessments could provide a more comprehensive understanding of the pathophysiology of SARS-CoV-2 infection and its short- and long-term consequences on the male reproductive system. Monitoring the baseline level of prolactin in male patients may help with the prognosis and clinical management of COVID-19 due to the high concentration of prolactin, which inhibits the HPG axis’ signaling [94]. E_2_ is an important sex hormone that originates from the conversion of testosterone conversion by aromatase, which is expressed mainly in testis adipose tissue, bone, and brain. Inflammation enhances aromatase activity and thus the conversion of testosterone to E_2_. Indeed, our meta-analysis demonstrates that E_2_ levels are significantly higher in patients with COVID-19 than in healthy control men. It needs to be further clarified whether this increase is a co-cause or consequence of COVID-19 and whether this has a prognostic impact. Some studies have found greater morbidity and mortality from COVID-19 in male patients than in females, so it can be assumed that estrogens have a protective role by modulating the inflammatory process. In this regard, a study conducted by Seeland et al. [95] suggested the protective role of estrogens against severe COVID-19 through the downregulation of the ACE2 receptor. The increase of estrogens could also be related to a decrease in the testosterone/E_2_ ratio for the transient state of primary hypogonadism developed as a consequence of the direct damage of the testicular epithelium by SARS-CoV-21. Although E_2_ could increase in patients with obesity, it must be considered that the present meta-analysis includes studies with homogenous populations in terms of age and BMI which did not show a significant difference between patients and controls, nor relevant heterogeneity that could alter the results. Thus, the increase in E_2_ is independent of the BMI.

The presence of impairment of testicular function during active infection is not surprising, since all infections in the acute phase are capable of interfering by various mechanisms with gonadotropin secretion and spermatogenesis. The current challenge is to understand whether SARS-CoV2 infection is able to depress testicular function in the long term, therefore independently of acute inflammatory processes. Our meta-analysis provides evidence on patients’ testicular function up to 80 days after infection and therefore has the potential to answer this question. To date, the longest time monitoring testicular function after recovery from COVID-19 was 90 days, a similar duration to that of the present meta-analysis. A recent uncontrolled multicenter study of eighty patients whose gonadal function was assessed 3 months after recovery from COVID-19 revealed an indirect and transient impairment of testicular function induced by SARS-CoV2 infection [80]. However, being uncontrolled, this study does not provide definite evidence of the impact of COVID-19 infection on testicular function after recovery, and further data are needed to clarify this issue.

Our analysis was conducted on a total of 1250 patients with an active or anamnestic infection of COVID-19 and 1232 healthy controls. A strength of our study is the selection criteria of the patient population since in all studies that we selected, the infection was confirmed by RT-PCR analysis on nasal or pharyngeal swabs and/or positive serology for IgA/IgA antibodies. Studies that included patients with presumed COVID-19 infection based on pulmonary computerized tomography results were not included. Therefore, our analysis proposes a homogeneous and representative sample of patients with SARS-CoV-2 infection.

### Limitations of the study

Our analysis has some limitations. Firstly, this is a short-term evaluation of patients with active or anamnestic COVID-19 infection (from baseline to up to 80 days from infection), thus it does not provide information on long-term follow-up and possible long-term consequences of COVID-19 infection on the male urogenital tract, on the recovery times from hypogonadism, and the possible consequences of protracted hormonal alterations.

Although our study population was homogeneous in terms of age and BMI, not all studies recorded clinical symptoms of the infection, associated comorbidities, or smoking habits. Furthermore, the interference of intercurrent pharmacological treatments (i.e., antivirals, antibiotics, corticosteroids, etc.) cannot be excluded. Some of the patients included in our study were aged from 57 to 65 years, an age for which a progressive decline in sperm parameters is already known in the literature [96–98]. Another limit is represented by the different timing of semen sample collection compared to the moment in which the infection was contracted. Some studies [99] reported collections performed during the acute symptomatic phase, while others [100] required collections about 70–80 days after infection and therefore during the convalescence phase. It has been suggested that semen analysis should be performed 3 months after infection to see changes in sperm quality once a new cycle of spermatogenesis is completed. Finally, some parameters of sperm quality, such as sperm DNA fragmentation (SDF), were not included in our study, as few studies have evaluated this parameter. Evaluation of SDF could open new insights into the understanding of SARS-CoV-2 pathophysiology and new perspectives on its implications for reproductive biology [101].

## Conclusion

The SARS-CoV-2 pandemic has spread worldwide since 2019, with varying degrees of severity and threat and a mortality rate that, in some countries, reaches up to 10% [102]. Controlling this epidemic is still challenging due to the emergence of new variants and the ability of the virus to spread and survive. Furthermore, research interest is moving toward understanding the long-term consequences of the virus on the various apparatuses of the human body. For this reason, we conducted the present meta-analysis to evaluate the effects of SARS-CoV-2 infection on male gonadal function, evaluating the hormonal profile and sperm parameters. The results demonstrated a negative effect of SARS-CoV-2 infection both on the hormonal and reproductive profiles, which were evaluated at up to 80 days from infection.

Even while recent research indicated that the negative effects of SARS-CoV-2 infection on semen quality may only be transient, the length of time after infection resolution before spermatogenesis is restored is another issue that is still up for debate [80]. A conclusion about these topics could be important for couples that are ART candidates, as it may be suggested to advise infertile couples to postpone parenthood research or ART procedures around 3 months after recovery from the infection to maximize their chances of getting pregnant, due to evidence of temporary alterations of sperm DNA integrity close to the recovery. Moreover, a conclusion regarding these issues could be crucial for men undergoing procedures to cryopreserve male gametes [103].

### Supplementary information

Supplementary Figure 1

Supplementary Figure 2

Supplementary Figure 3

Supplementary Figure 4

Supplementary Figure 5

Supplementary Figure 6

Supplementary Figure 7

Supplementary Figure 8

Supplementary Figure 9

Supplementary Figure 10

Supplementary Figure 11

Supplementary Figure 12

Supplementary Figure 13

Supplementary Figure 14

Legend to the Supplementary Figures

## Data Availability

Data will be made available to the editors of the journal for review or query upon request.
