# Supplementary figures and images for: Impact of COVID-19 on testicular function: a systematic review and meta-analysis
Source: Endocrine. 2024 Feb 12;85(1):44–66. doi: 10.1007/s12020-024-03705-7 (PMC11246276; doi:10.1007/s12020-024-03705-7)

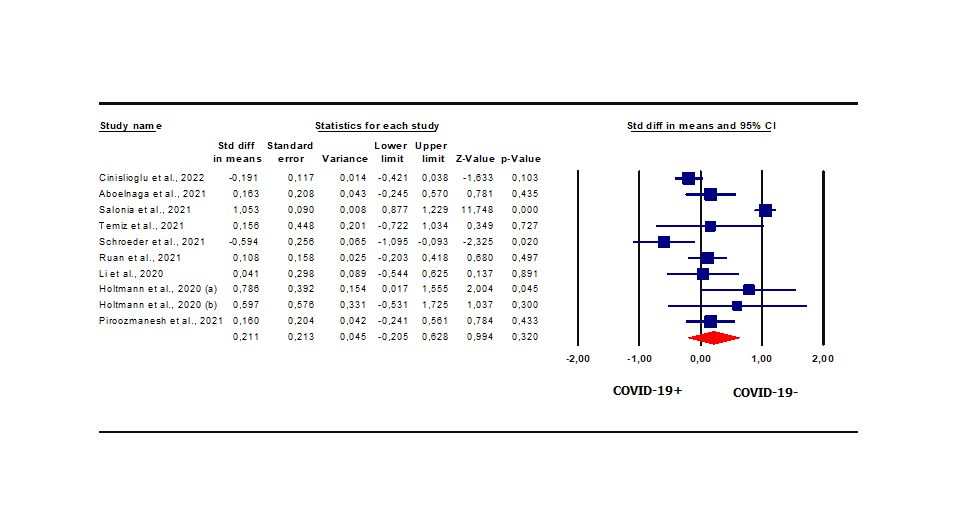

Supplement: Supplementary file 1 — Supplementary Figure 1 [file 12020_2024_3705_MOESM1_ESM.tif]

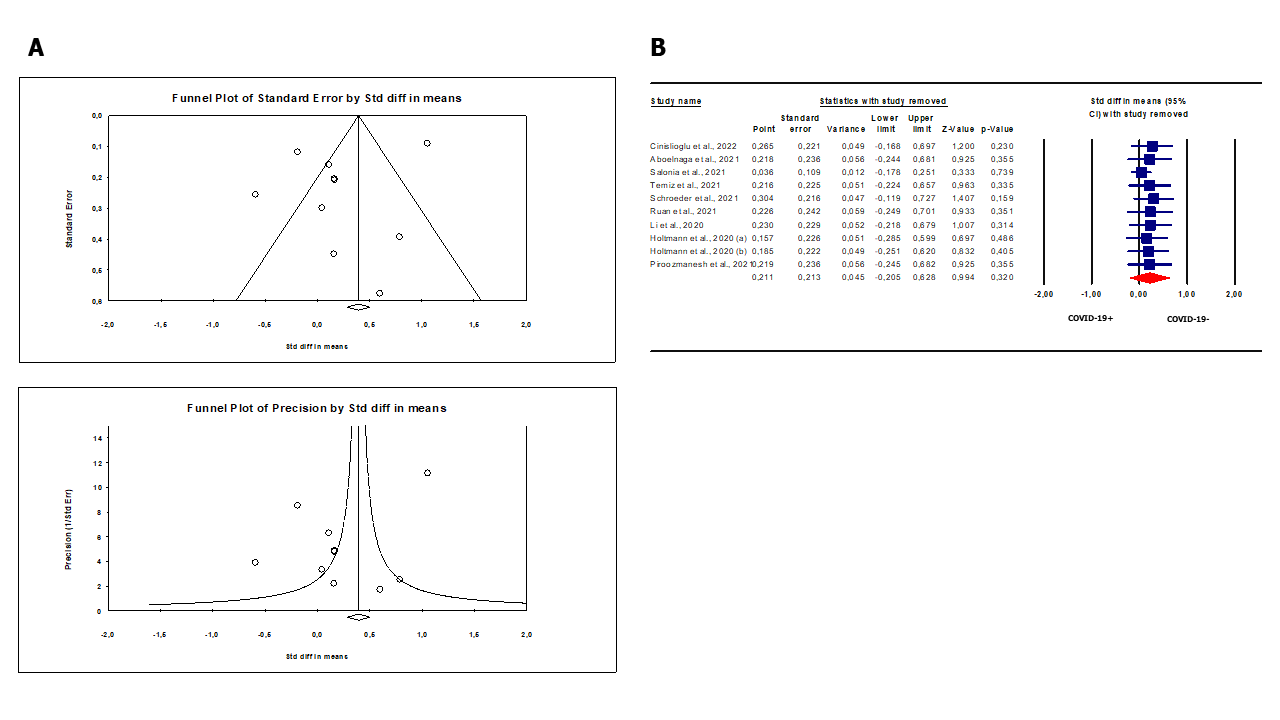

Supplement: Supplementary file 2 — Supplementary Figure 2 [file 12020_2024_3705_MOESM2_ESM.tif]

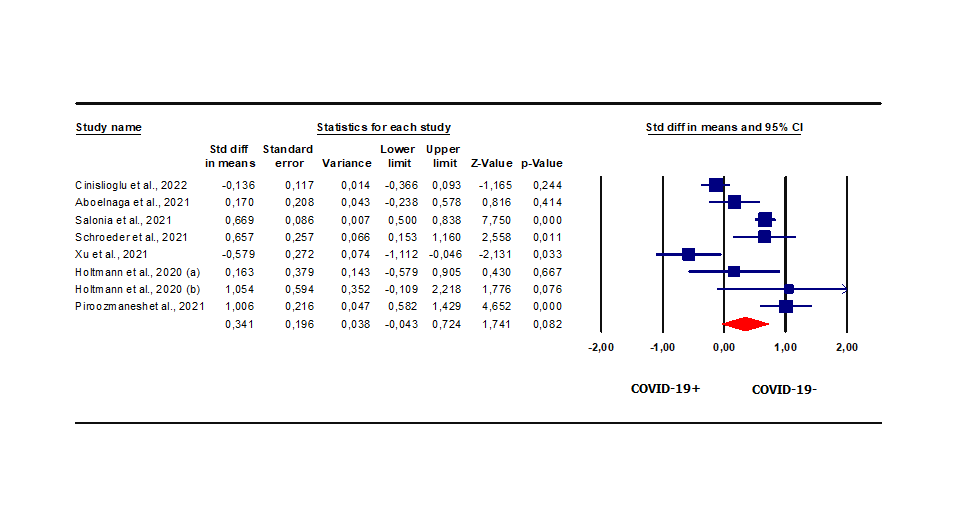

Supplement: Supplementary file 3 — Supplementary Figure 3 [file 12020_2024_3705_MOESM3_ESM.tif]

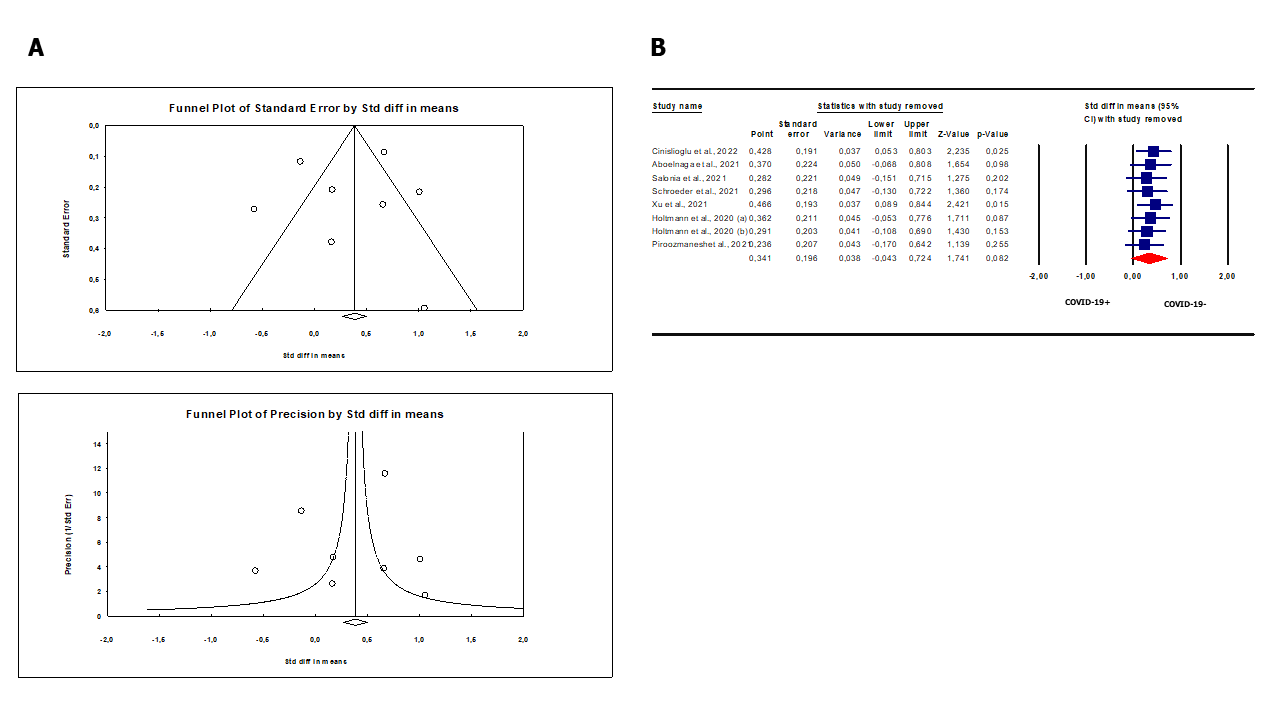

Supplement: Supplementary file 4 — Supplementary Figure 4 [file 12020_2024_3705_MOESM4_ESM.tif]

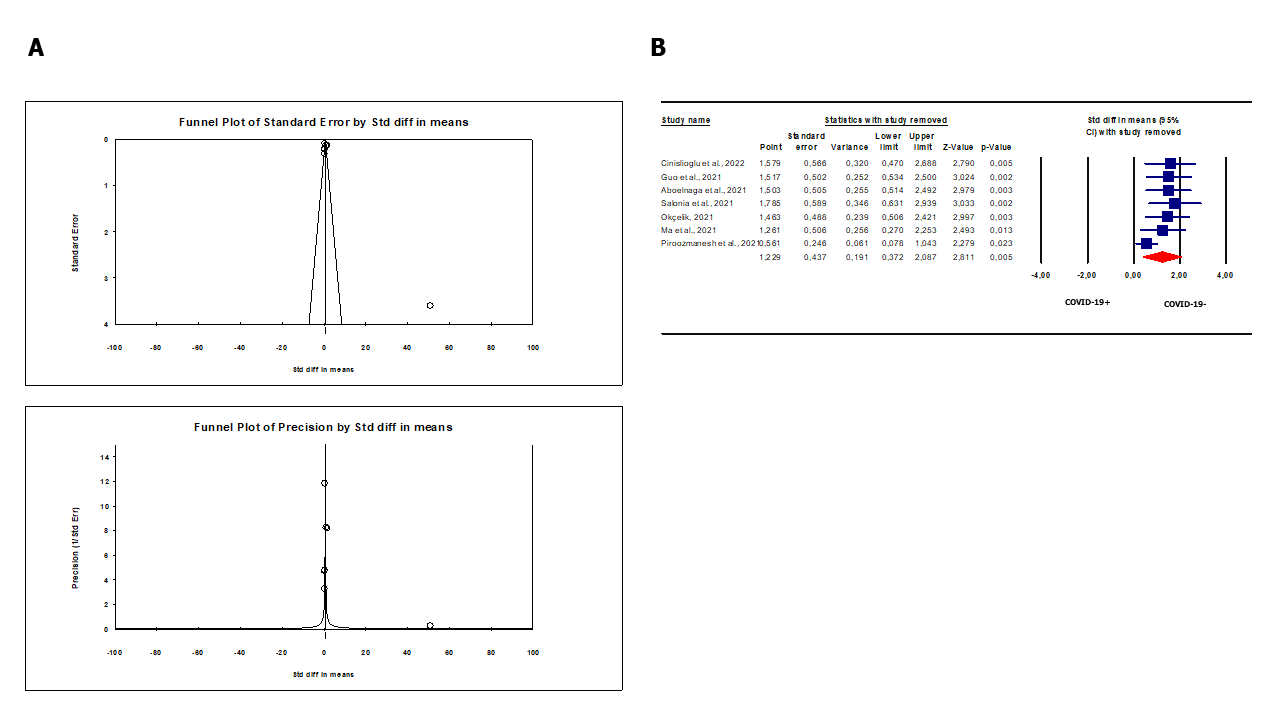

Supplement: Supplementary file 5 — Supplementary Figure 5 [file 12020_2024_3705_MOESM5_ESM.tif]

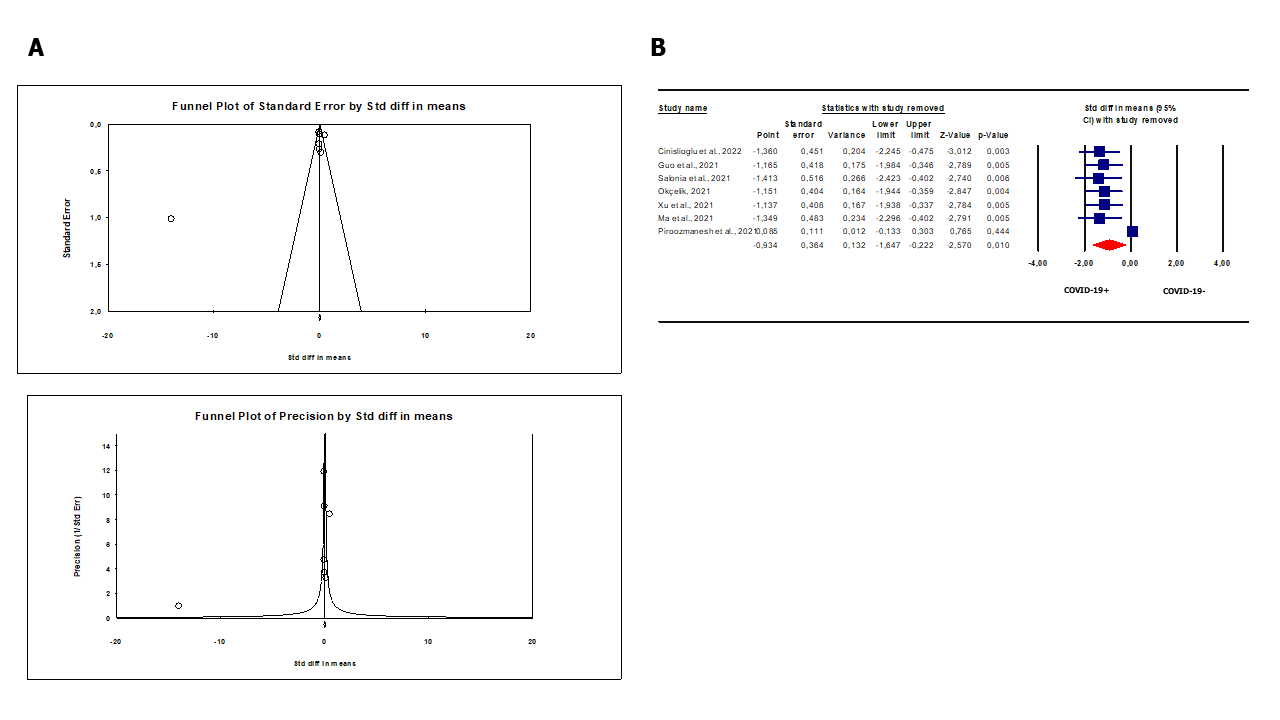

Supplement: Supplementary file 6 — Supplementary Figure 6 [file 12020_2024_3705_MOESM6_ESM.tif]

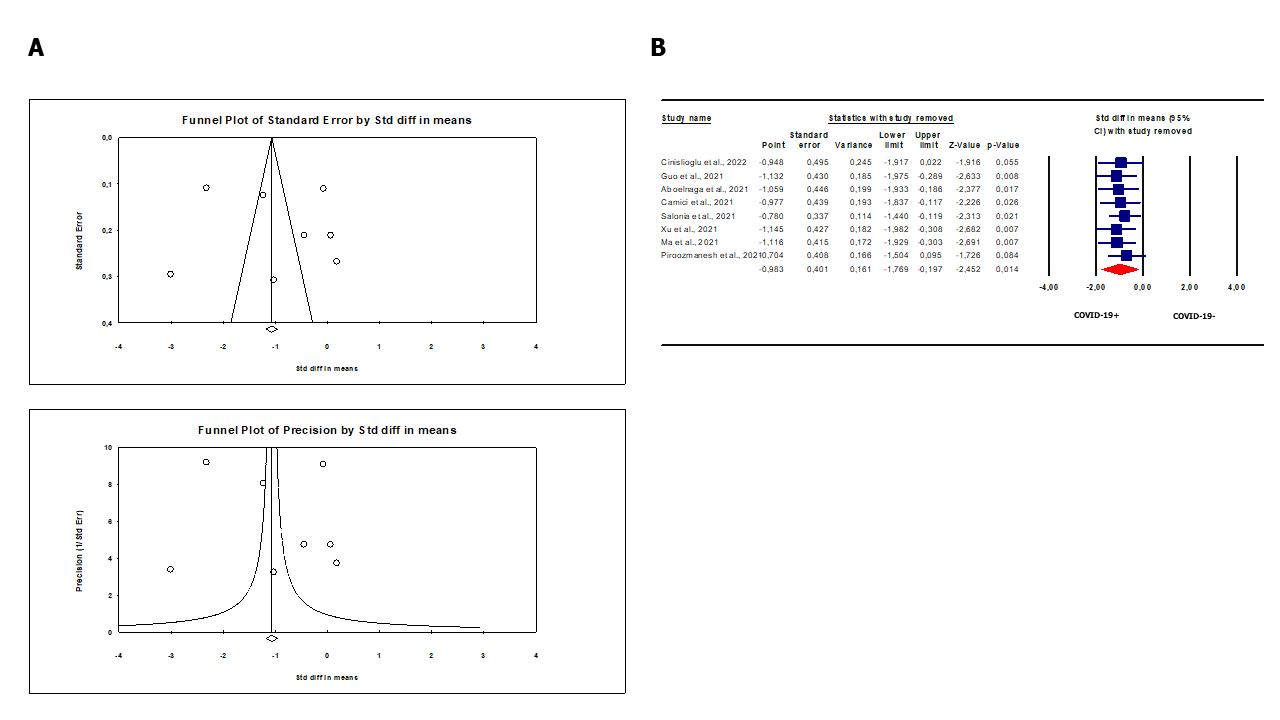

Supplement: Supplementary file 7 — Supplementary Figure 7 [file 12020_2024_3705_MOESM7_ESM.tif]

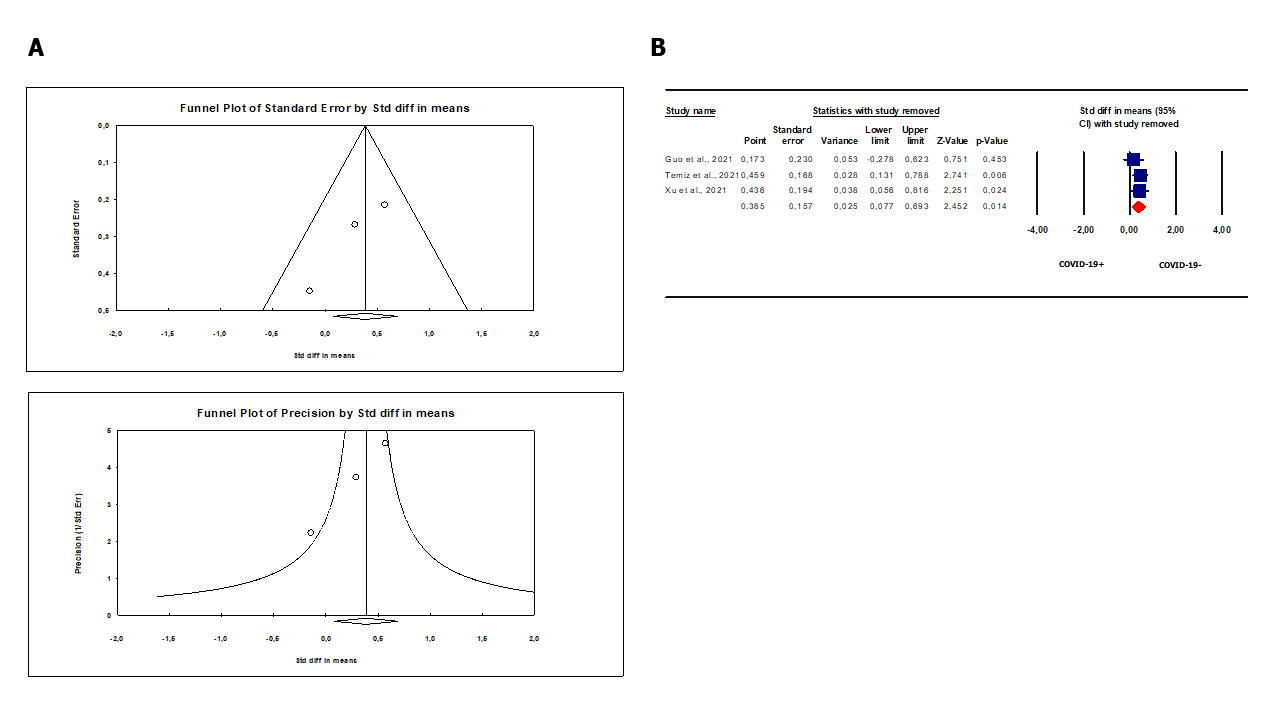

Supplement: Supplementary file 8 — Supplementary Figure 8 [file 12020_2024_3705_MOESM8_ESM.tif]

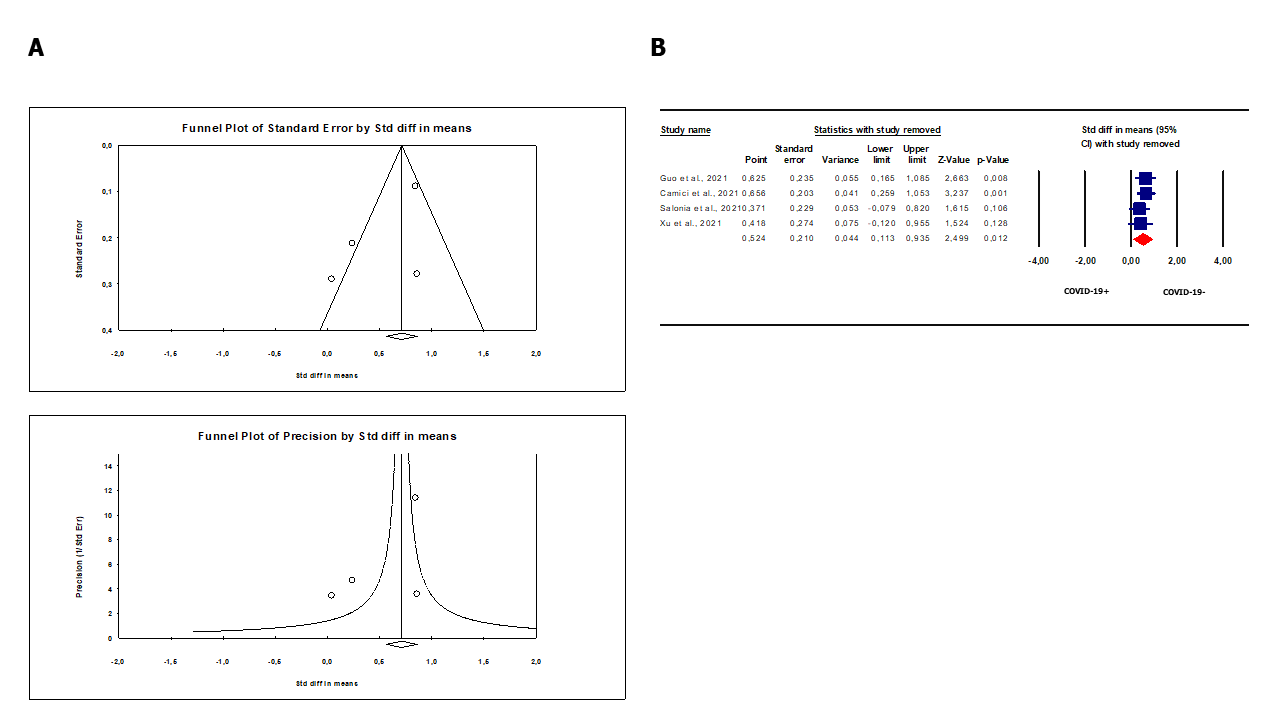

Supplement: Supplementary file 9 — Supplementary Figure 9 [file 12020_2024_3705_MOESM9_ESM.tif]

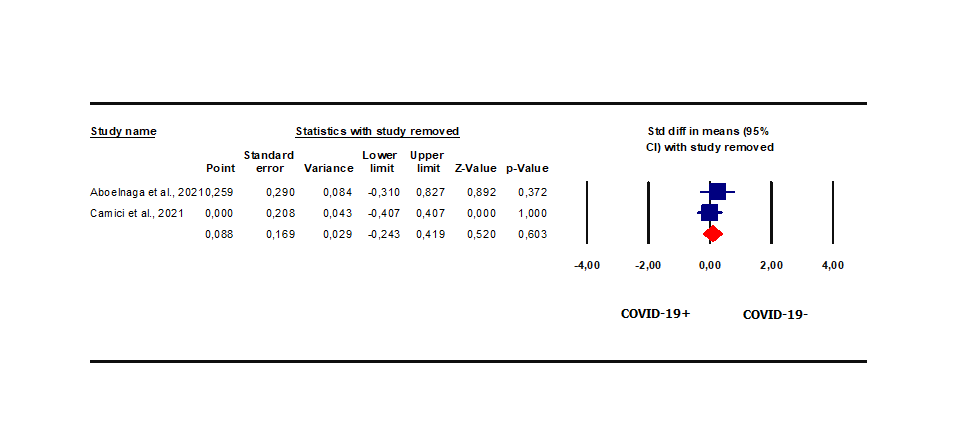

Supplement: Supplementary file 10 — Supplementary Figure 10 [file 12020_2024_3705_MOESM10_ESM.tif]

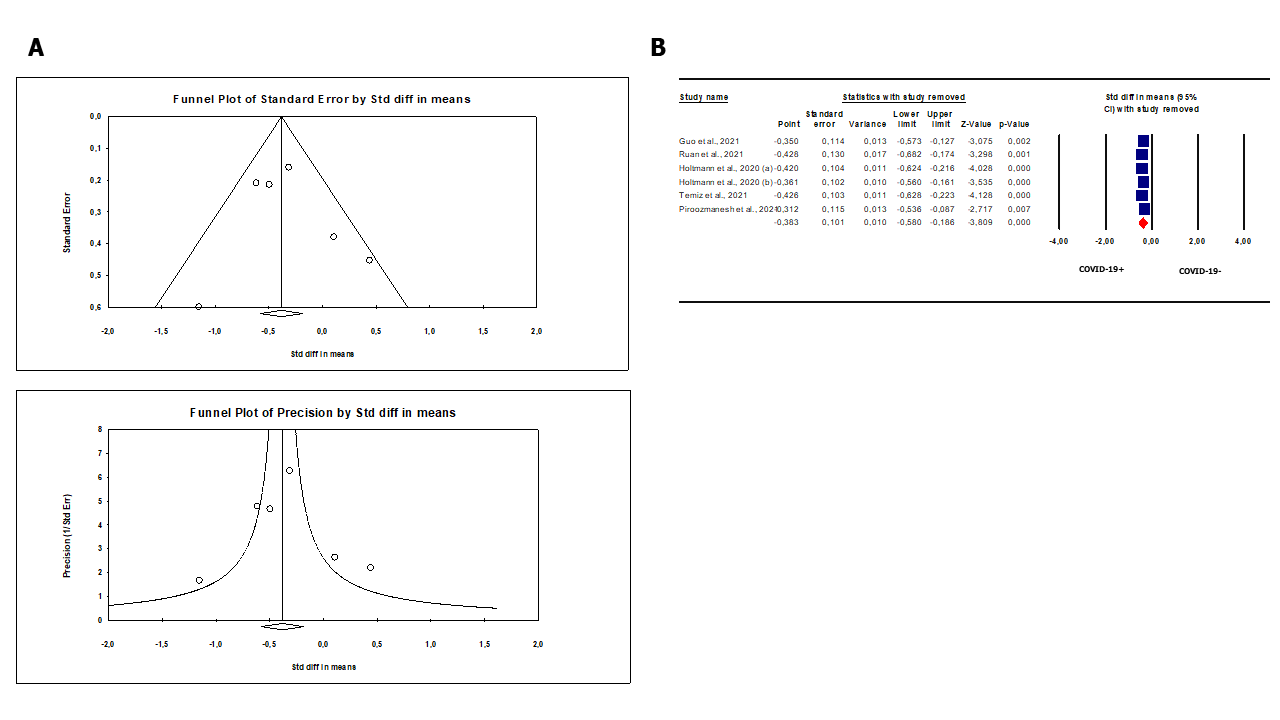

Supplement: Supplementary file 11 — Supplementary Figure 11 [file 12020_2024_3705_MOESM11_ESM.tif]

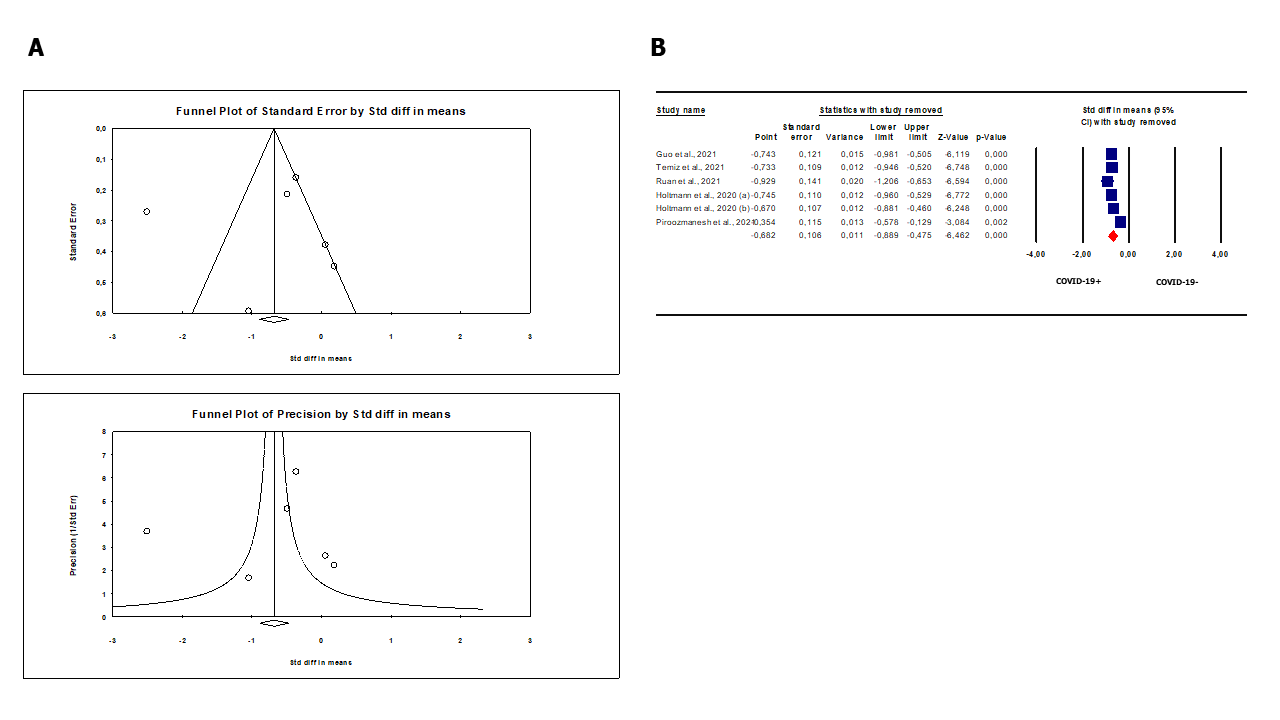

Supplement: Supplementary file 12 — Supplementary Figure 12 [file 12020_2024_3705_MOESM12_ESM.tif]

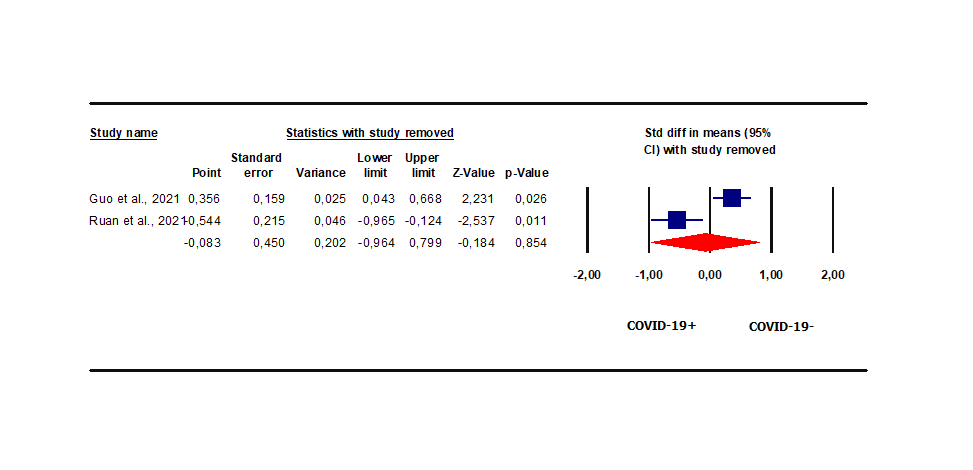

Supplement: Supplementary file 13 — Supplementary Figure 13 [file 12020_2024_3705_MOESM13_ESM.tif]

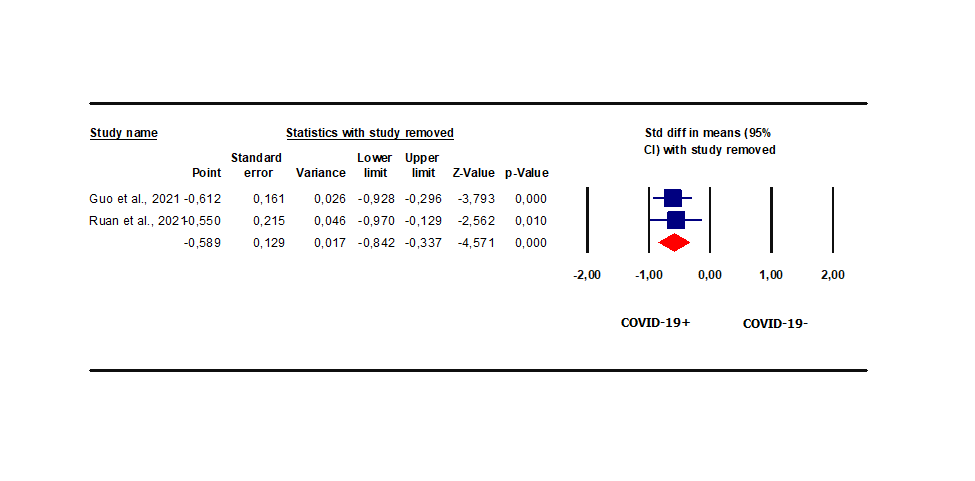

Supplement: Supplementary file 14 — Supplementary Figure 14 [file 12020_2024_3705_MOESM14_ESM.tif]
