# Supplementary material for: Impact of COVID-19 on testicular function: a systematic review and meta-analysis
Source: Endocrine. 2024 Feb 12;85(1):44–66. doi: 10.1007/s12020-024-03705-7 (PMC11246276; doi:10.1007/s12020-024-03705-7)
Supplement: Supplementary file 15 — Legend to the Supplementary Figures [file 12020_2024_3705_MOESM15_ESM.docx]

**Legend to the Supplementary Figures**

**Supplementary Figure 1.** Forest plot of the age in patients and controls.

**Supplementary Figure 2.** Funnel plot (A) and sensitivity analysis (B) of the age in patients and controls.

**Supplementary Figure 3.** Forest plot of the body mass index in patients and controls.

**Supplementary Figure 4.** Funnel plot (A) and sensitivity analysis (B) of the body mass index in patients and controls.

**Supplementary Figure 5.** Funnel plot (A) and sensitivity analysis (B) of the luteinizing hormone in patients and controls.

**Supplementary Figure 6.** Funnel plot (A) and sensitivity analysis (B) of the follicle-stimulating hormone in patients and controls.

**Supplementary Figure 7.** Funnel plot (A) and sensitivity analysis (B) of total testosterone in patients and controls.

**Supplementary Figure 8.** Funnel plot (A) and sensitivity analysis (B) of prolactin in patients and controls.

**Supplementary Figure 9.** Funnel plot (A) and sensitivity analysis (B) of 17β-estradiol in patients and controls.

**Supplementary Figure 10.** Sensitivity analysis of sex hormone binding globulin in patients and controls.

**Supplementary Figure 11.** Funnel plot (A) and sensitivity analysis (B) of sperm concentration in patients and controls.

**Supplementary Figure 12.** Funnel plot (A) and sensitivity analysis (B) of total sperm count in patients and controls.

**Supplementary Figure 13.** Sensitivity analysis of progressive sperm motility in patients and controls.

**Supplementary Figure 14.** Sensitivity analysis of total sperm motility in patients and controls.
